# Enhanced mitochondrial fission suppresses signaling and metastasis in triple-negative breast cancer

**DOI:** 10.1186/s13058-020-01301-x

**Published:** 2020-06-05

**Authors:** Brock A. Humphries, Alyssa C. Cutter, Johanna M. Buschhaus, Yu-Chih Chen, Tonela Qyli, Dilrukshika S. W. Palagama, Samantha Eckley, Tanner H. Robison, Avinash Bevoor, Benjamin Chiang, Henry R. Haley, Saswat Sahoo, Phillip C. Spinosa, Dylan B. Neale, Jagadish Boppisetti, Debashis Sahoo, Pradipta Ghosh, Joerg Lahann, Brian D. Ross, Eusik Yoon, Kathryn E. Luker, Gary D. Luker

**Affiliations:** 1 Center for Molecular Imaging, Department of Radiology, University of Michigan, 109 Zina Pitcher Place, Ann Arbor, MI 48109 USA; 2 Department of Biomedical Engineering, University of Michigan, 109 Zina Pitcher Place, Ann Arbor, MI 48109 USA; 3 Department of Electrical Engineering and Computer Science, University of Michigan, Ann Arbor, MI USA; 4 Comprehensive Cancer Center, University of Michigan, Ann Arbor, MI USA; 5 Forbes Institute for Cancer Discovery, University of Michigan, Ann Arbor, MI USA; 6 Unit for Laboratory Medicine, University of Michigan, Ann Arbor, MI USA; 7 Department of Chemical Engineering, University of Michigan, Ann Arbor, MI USA; 8 Biointerfaces Institute, University of Michigan, Ann Arbor, MI USA; 9 Department of Pediatrics, Department of Computer Science and Engineering, Jacob’s School of Engineering, Rebecca and John Moore Comprehensive Cancer Center, University of California San Diego, La Jolla, CA USA; 10 Department of Medicine, Department of Cellular and Molecular Medicine, Rebecca and John Moore Comprehensive Cancer Center, Veterans Affairs Medical Center, University of California San Diego, La Jolla, CA USA; 11 Biointerfaces Institute, Departments of Chemical Engineering, Materials Science and Engineering, Biomedical Engineering, and Macromolecular Science and Engineering, University of Michigan, Ann Arbor, MI USA; 12 Department of Microbiology and Immunology, University of Michigan, 109 Zina Pitcher Place, Ann Arbor, MI 48109 USA

**Keywords:** Triple-negative breast cancer, ERK, Akt, Fluorescence microscopy, Mitochondrial fission, Mitochondrial fusion, Metastasis

## Abstract

**Background:**

Mitochondrial dynamics underlies malignant transformation, cancer progression, and response to treatment. Current research presents conflicting evidence for functions of mitochondrial fission and fusion in tumor progression. Here, we investigated how mitochondrial fission and fusion states regulate underlying processes of cancer progression and metastasis in triple-negative breast cancer (TNBC).

**Methods:**

We enforced mitochondrial fission and fusion states through chemical or genetic approaches and measured migration and invasion of TNBC cells in 2D and 3D in vitro models. We also utilized kinase translocation reporters (KTRs) to identify single cell effects of mitochondrial state on signaling cascades, PI3K/Akt/mTOR and Ras/Raf/MEK/ERK, commonly activated in TNBC. Furthermore, we determined effects of fission and fusion states on metastasis, bone destruction, and signaling in mouse models of breast cancer.

**Results:**

Enforcing mitochondrial fission through chemical or genetic approaches inhibited migration, invasion, and metastasis in TNBC. Breast cancer cells with predominantly fissioned mitochondria exhibited reduced activation of Akt and ERK both in vitro and in mouse models of breast cancer. Treatment with leflunomide, a potent activator of mitochondrial fusion proteins, overcame inhibitory effects of fission on migration, signaling, and metastasis. Mining existing datasets for breast cancer revealed that increased expression of genes associated with mitochondrial fission correlated with improved survival in human breast cancer.

**Conclusions:**

In TNBC, mitochondrial fission inhibits cellular processes and signaling pathways associated with cancer progression and metastasis. These data suggest that therapies driving mitochondrial fission may benefit patients with breast cancer.

## Background

As hubs for metabolism and biosynthesis, mitochondria adapt to meet energetic and anapleurotic demands of malignant transformation, unregulated proliferation, and tumor progression [1, 2]. Mitochondria transition between fused and fissioned states mediated by mitofusins 1 and 2 (Mfn1/2) with optic atrophy 1 (Opa1) for fusion and dynamin-related protein 1 (Drp1) for fission [3]. Fused mitochondria support efficient transfer of contents within the organelle and coupling of complexes in electron transport to facilitate oxidative phosphorylation. Shifts between fission and fused states allow cells to adapt to changes in available nutrients and energy demands [4]. Inputs from multiple signaling pathways modulate mitochondrial fission and fusion, providing control points that allow mitochondrial phenotypes to sense and respond to environmental conditions.

Not only does mitochondrial morphology regulate metabolic plasticity [5], but recent work also points to broader functions for fission and fusion in signal transduction [6, 7]. Mitochondria serve as scaffolds for localization and assembly of signaling molecules and complexes. Many MAP kinases and Akt localize to mitochondria to regulate cell signaling and behaviors in normal and malignant settings [8–12]. Mitochondria also regulate levels of metabolic signaling molecules, such as reactive oxygen species (ROS) and ATP. Recent evidence suggests that mitochondrial morphology transitions control the release of ROS and ATP [13, 14], directly impacting cell function and survival. Therefore, functions of mitochondria as regulators of cell signaling suggest the balance between mitochondrial fission and fusion controls tumor progression and represents potential targets for therapy.

Our previous work demonstrated that phosphatidylserine decarboxylase (PISD), a mitochondrial enzyme that converts phosphatidylserine (PS) to phosphatidylethanolamine (PE), drives mitochondrial fission [15]. Building on this finding, here, we investigate effects of mitochondrial morphology on breast cancer metastasis. We discover that enforcing mitochondrial fission through genetic and chemical methods reduced metastatic potential of triple-negative breast cancer cells. Furthermore, we identify inhibitory effects of mitochondrial fission on Akt and ERK signaling in cell-based assays and mouse models of breast cancer. Enforcing mitochondrial fusion reverses these phenotypes, increasing metastasis and signaling. We show that opposing effects of mitochondrial fission and fusion extend to patients with breast cancer, where improved survival correlates with high levels of fission genes and low levels of genes driving mitochondrial fusion. Overall, these data demonstrate the regulation of breast cancer metastasis by mitochondrial dynamics and, for the first time, establish a direct effect of mitochondrial morphology on Akt and ERK signaling in breast cancer.

## Methods

### Cell culture

We purchased MDA-MB-231 cells from the ATCC (Manassas, VA) and cultured cells in Dulbecco’s modified Eagle medium (DMEM) supplemented with 10% fetal bovine serum (FBS), 1% penicillin/streptomycin (Pen/Strep) (Thermo Fisher Scientific, Waltham, MA), and 1% GlutaMAX (Thermo Fisher Scientific, Waltham, MA). We obtained SUM159 cells from Dr. Stephen Ethier (now at The Medical University of South Carolina, Charleston, SC) and cultured cells in F-12 media supplemented with 10% fetal bovine serum, 1% Pen/Strep, 1% glutamine, 5 μg/mL hydrocortisone, and 1 μg/mL insulin. We authenticated all cells by analysis of short tandem repeats and characterized cells as free of *Mycoplasma* at the initial passage. We used all cells within 3 months after resuscitation and maintained all cells at 37 °C in a humidified incubator with 5% CO_2_.

### Vectors and cell lines

We used MDA-MB-231 and SUM159 cells stably expressing PISD-mCherry as described in our recent study [15] for initial experiments. Plasmid pLV-mito-GFP was a gift from Pantelis Tsoulfas (Addgene plasmid #44385), and we also used this mitochondrial targeted GFP (Mito-GFP) to visualize mitochondrial morphology in our initial experiments as described previously [15, 16].

To identify the activity of ERK and Akt, we used the kinase translocation reporter (KTR) for ERK and Akt as previously described [17, 18]. This KTR construct contains H2B fused to mCherry (H2B-mCherry), the Akt-KTR reporter (Aquamarine), the ERK-KTR reporter (mCitrine), and a puromycin selection marker all separated by P2A linker sequences cloned into the Piggyback transposon vector as described previously (pHAEP) [18]. To stably express PISD in cells with the KTR construct, we generated a construct with unlabeled PISD and a hygromycin selection marker separated by a P2A sequence (PISD-hygromycin). To visualize mitochondria concurrently in cells with the KTR construct, we replaced GFP in the Mito-GFP plasmid with mTagBFP2 (Mito-BFP) and added a neomycin selection marker (Mito-BFP-neomycin). For stable expression of Drp1, we used the pEYFP-C1-Drp1 plasmid [19], a gift from Richard Youle (Addgene plasmid #45160; http://n2t.net/addgene:45160; RRID: Addgene_45160). We cloned the Drp1 reading frame from this plasmid into the pLVX vector and added a hygromycin selection marker separated by a P2A sequence (Drp1-hygromycin). We produced recombinant lentiviral vectors for PISD-hygromycin, Mito-BFP-neomycin, and Drp1-hygromycin as previously described [20].

We first generated cells stably expressing click beetle green luciferase (SUM159-CBG and MDA-MB-231-CBG) as described previously through selection with blasticidin [21]. We next transfected cells with the Piggyback transposon vector containing both KTRs (pHAEP) using FuGENE HD (Promega). We selected stably expressing cells using puromycin and confirmed expression by fluorescence as previously described [17]. We next transduced cells (SUM159-CBG-pHAEP and MDA-MB-231-CBG-pHAEP) with the Mito-BFP-neomycin lentiviral vector and selected cells using neomycin, generating our wild-type (WT) cells (SUM159- and MDA-MB-231-CBG-pHAEP Mito-BFP-neomycin). Next, we transduced cells with either PISD- or Drp1-hygromycin and selected for stable expression using hygromycin. For cells expressing only Mito-BFP only cells, we transduced cells already expressing CBG with the Mito-BFP construct and selected using neomycin. After selection, we transduced cells with PISD-hygromycin and selected for a stably expressing population. For cells with only PISD or Drp1, after we added and selected for CBG stably expressing cells, we introduced the PISD- or Drp1-hygromycin construct into cells already stably expressing CBG and then selected with hygromycin.

### qRT-PCR

To analyze levels of PISD, we performed qRT-PCR for PISD and β-actin using SYBR Green detection as described previously [22]. Primers for PISD (Sigma Aldrich) were 5′-CTCCATTCGCATCTACTTTG-3′ and 5′-AGCTGAAGTCATTGTAGGAG-3′ and β-actin 5′-TGTACGTTGCTATCCAGGCTGTGC-3′ and 5′-CGGTGAGGATCTTCATGAGGTAGTC-3′.

### Lipid supplementation

We purchased l-α-lysophosphatidylethanolamine (LPE, cat 860081) from Avanti Polar Lipids Inc. (Alabaster, AL) and prepared a 50-mM stock as described previously [23].

### Mitochondrial morphology assays

To visualize mitochondrial morphology, we seeded 2 × 10^4^ cells onto 35-mm dishes with a 20-mm glass bottom (Cellvis, Mountain View, CA) in FluoroBrite DMEM media (ThermoFisher Scientific, cat. A1896701), containing 10% FBS, 1% Pen/Strep, 1% GlutaMAX, and 1% Sodium Pyruvate (Thermo Fisher Scientific, Waltham, MA). Two and 3 days after seeding, we changed media to FluoroBrite DMEM media as described above containing either LPE (50 μM) to drive fragmentation, leflunomide (Cayman Chemical Company, cat: 14860; 50 μM) to drive fusion, or vehicle control. For visualization, we acquired images on an Olympus IX73 microscope with a DP80 CCD camera (Olympus) at the time points indicated in the figure.

### Migration assays

To determine effects of mitochondrial morphology on migration, after treatment of cells with lipids as described in the “Mitochondrial morphology assays” section, we loaded cells into our microfluidic migration device as previously described and quantified the distance each cell moved within the device [15].

To study the effects of PISD on cell migration in a complex 3D environment, we used tissue-engineering constructs consisting of a SU8 construct coated with networks of engineered fibrillar fibronectin [24]. We seeded 5 × 10^4^ SUM159-CBG-WT or PISD cells stably expressing pHAEP onto the construct while rotating for 1 h at 37 °C. After 1 h, we washed the construct with PBS and placed it in a 35-mm dish with a 20-mm glass bottom. We covered the construct with 2 mL of FluoroBrite DMEM containing the same additives as described in the “Cell culture” section and placed the dish in the incubator for 3 h. After 3 h, we imaged the dish every 20 min for 3 h on an Olympus FVMPE-RS upright two-photon microscope using settings as described previously [18]. We identified and tracked cells using custom MATLAB code that tracked the H2B-mCherry nucleus of cells on the construct.

### Lipid extraction

We extracted total lipids from SUM159-WT and PISD cells based on a modified protocol described previously [25]. We homogenized SUM159-WT and PISD pellets containing 1 × 10^7^ cells in 0.75 mL of methanol. We transferred the homogenate into 1.5-mL glass vials and added 0.75 mL of chloroform. We sonicated the mixture for 30 min and then centrifuged the mixture at 9000 rpm for 5 min at RT, transferring the liquid layer to another tube. We performed an extraction on the remaining solid phase again using a 2:1 (v/v) chloroform to methanol solution, followed by sonication for 30 min, centrifugation, and then combining the two extracted liquid phases. We repeated this procedure twice more, combining the extracted lipids in the liquid phase each time (total of 4 extractions).

### Mass spectrometry

For MALDI mass spectrometry (MS), we re-dissolved the lipid extract in 2 mL of chloroform to methanol (3:2 v/v) containing 1 mM NaCH_3_COO. We mixed equal amounts of the re-dissolved lipid extract and the matrix solution (10 mg/mL 2,5-dihydroxybenzoic acid (DHB) in acetonitrile to water (70:30% v/v)) and spotted 1 μL of the mixture on a stainless steel MALDI-plate. We dried the sample in a dessicator and analyzed the spot by MALDI-MS and MS/MS in positive ion modes using a MALDI-TOF-MS instrument (SYNAPT G2-Si, Waters US, Milford, MA). We acquired and analyzed data using Masslynx (Waters, Milford, MA). We identified lipids using MALDI-MS/MS and the LIPID MAPS database for reference (https://www.lipidmaps.org/).

For MALDI imaging mass spectrometry (IMS), we injected 1 × 10^6^ SUM159- and MDA-MB-231-WT and PISD cells orthotopically into the fourth mammary fat pad of 16–23-week-old female NSG mice. After tumors reached ~ 1 cm in diameter, we removed tumors, mounted tissue on a cryotome tissue block using Shandon M-1 Embedding matrix (ThermoFisher, 1310TS), and placed samples on dry ice. We then stored the sample at − 80 °C until sectioning. We cryosectioned 10-μm sections of each tumor and placed them on clean, untreated microscope slides. We dried slides under vacuum for 30 min, and then we sprayed the samples with 40 mg/mL DHB in 1:1 water to methanol (v/v) containing 0.1% trifluoroacetic acid using a HTX M5 matrix sprayer (HTX Technologies, Chapel Hill, NC). We collected IMS data in positive ion mode and analyzed data using HDImaging software (Waters, Milford, MA).

### ATP, mitochondrial mass, and membrane potential analysis

We analyzed effects of PISD on cellular ATP levels using an ATP colorimetric assay kit (Sigma Aldrich, cat. MAK190) following the manufacturer’s instructions. To determine effects of LPE and leflunomide on mitochondrial mass and membrane potential, we utilized MitoTracker® Green FM (Invitrogen, M7514) and MitoProbe™ JC-1 Assay Kit (Thermo Fisher, M34152) as per the manufacturer’s protocols, respectively [15].

### Three-dimensional assays

We formed spheroids as previously described [21], co-culturing WT or PISD cells expressing mitochondrially targeted GFP with human mammary fibroblasts (HMFs) expressing mCherry (1:1 ratio of cancer cells to HMFs). We embedded spheroids in fibrin gels (final concentration 4 mg/mL fibrin, 2.5 U/mL thrombin, and 0.01 U/mL aprotinin [26]) and covered in media containing aprotinin (0.01 U/mL) and LPE (50 μM) or leflunomide (50 μM). On the day after embedding (day 1), we added LPE (50 μM) and leflunomide (50 μM) to fresh culture media covering gels. Two days after seeding spheroids in fibrin gels, we imaged spheroids using two-photon microscopy. We quantified the radius and points on the perimeter of the spheroid using custom MATLAB code [17].

### Kinase translocation reporter

To quantify the activation of both ERK and Akt kinases in single cells, we used previously validated kinase translocation reporters (KTR). KTRs measure activities of ERK and Akt by utilizing a known downstream substrate specific for each kinase fused to a fluorescent protein (citrine and aquamarine for ERK and Akt, respectively). Phosphorylation and dephosphorylation of a KTR reversibly shifts localization from cytoplasm to nucleus, respectively [27, 28]. For 2D signaling experiments, we seeded 1.2 × 10^5^ (bulk) or 1.0 × 10^5^ (single cell mitochondrial analysis) MDA-MB-231- and 8.5 × 10^4^ (bulk) or 8.0 × 10^4^ (single cell mitochondrial analysis) SUM159-WT or PISD cells into 35-mm dishes with a 20-mm glass bottom (Cellvis, Mountain View, CA) in FluoroBrite DMEM media (ThermoFisher Scientific, A1896701). After 2 days, we changed medium to Fluorobrite DMEM containing either LPE (50 μM), leflunomide (50 μM), or vehicle control. The following day, we changed media to low (1%) serum FluoroBrite DMEM media containing either LPE (50 μM), leflunomide (50 μM), or vehicle control. After overnight incubation, we treated cells with either serum (10%), EGF (50 ng/mL, R&D Systems, Minneapolis, MN), or vehicle control and acquired images at the times listed in the figure. For bulk signaling experiments, we presented changes in activation of Akt and ERK as the earth mover’s distance (EMD) relative to the initial time point (0 min) [29, 30]. The EMD is a mathematical measure of the distance between two samples. It thinks of two histogram datasets as piles of dirt in multivariate space, and the EMD score is the minimum cost of moving one pile into the other. The higher the EMD score, the greater the difference between the two histograms [30]. There is no *p* value associated with the EMD score, as it is a statistical test itself.

### Kinase translocation reporter and mitochondrial morphological analysis

We analyzed kinase translocation reporter images for activation of Akt and ERK and identified mitochondrial morphology using an adaptive thresholding method in MATLAB as described previously [17, 18]. For individual fluorescence microscopy images of mitochondria, we used MATLAB to skeletonize the mitochondria, measure total pixel area and major axis length using the regionprops command, and assign each mitochondrion a pixel ID. We separated mitochondria into fused or fissioned groups by area and major axis length using a user-defined cutoff that we applied to every cell group analyzed in each experiment. We categorized an identified mitochondrion that had total area/majoraxislength > 2 as networked. For dynamic mitochondrial analysis in cells expressing KTRs, we first took the two-photon microscopy images and made a Z-stack, correcting for any dish movement, and analyzed the mitochondria as described above. We will provide MATLAB code upon request.

### Immunofluorescence staining

In addition to KTRs, we identified changes in active and total ERK and Akt kinases using immunofluorescence staining. We seeded cells as described in the “Kinase translocation reporter” section and stained cells in response to serum at the time points indicated in the figure. We used the following primary antibodies from Cell Signaling Technologies (Danvers, MA, USA): anti-phospho-Akt (Ser473) (cat. 4058), anti-pan-Akt (cat. 2920), anti-phospho-ERK (Thr202/Tyr204) (cat. 4370), and anti-pan-ERK (cat. 4696). We detected primary antibodies with fluorescent secondary antibodies from Jackson ImmunoResearch Laboratores Inc. (West Grove, PA, USA): anti-rabbit AlexaFluor 488 (cat. 111-545-003) and anti-mouse AlexaFluor 594 (cat. 115-585-003).

### Laurdan staining

We analyzed differences in membrane order among cells with the fluorescent dye laurdan. After treating various cells as described in the “Mitochondrial morphology assays” section, we stained cells (~ 70% confluency) with 5 μM Laurdan (Cayman Chemical, cat. 19706) as previously described [31] and imaged cells using two-photon microscopy (excitation 800 nm, emission 400–460 nm and 470–530 nm) [32]. We calculated general fluorescence polarization (GP) of laurdan using custom MATLAB code as described previously [31].

### Mouse studies

The University of Michigan IACUC approved all animal procedures (protocol 00008822). The animals used in this study received humane care in compliance with the principles of laboratory animal care formulated by the National Society for Medical Research and Guide for the Care and Use of Laboratory Animals prepared by the National Academy of Sciences and published by the National Institute of Health (Publication no NIH 85-23, revised 1996).

We established orthotopic tumor xenografts in the fourth inguinal mammary fat pads of 32-week-old female NSG mice as described previously [22], implanting 2 × 10^5^ SUM159- or MDA-MB-231-CBG-pHAEP Mito-BFP (WT) or PISD stably expressing cells.

To generate systemic metastases, we injected 1 × 10^5^ MDA-MB-231-WT or PISD breast cancer cells or SUM159 cells expressing different mitochondrial morphologies directly into the left ventricle of the heart of 6–8-week-old female NSG mice (Jackson Laboratory, Bar Harbor, ME, USA) as previously described [33]. All injected cells stably expressed click beetle green (CBG) luciferase for bioluminescence imaging. We quantified metastatic burden over 20–21 days after injection by bioluminescence imaging as previously described [21]. Due to spontaneous mortality immediately following intracardiac injection (one in the WT group and three in the PISD group), we present data only for mice that reached the experimental endpoint. Immediately after euthanizing mice, we imaged lung metastases ex vivo by two-photon microscopy. We used the excitation and emission filters described in our previous publication and quantified activation of Akt and ERK using custom MATLAB code [18]. For analysis of metastases to the bone marrow from intracardiac injection, we flushed the bone marrow with PBS from the femur and tibia and measured bioluminescence from cancer cells as described previously [34].

To identify effects of PISD on bone metastasis directly, we injected 2.5 × 10^4^ MDA-MB-231-WT or PISD cells into the left femoral artery of female 15–18-week-old NSG mice as previously described [35]. We tracked tumor progression by bioluminescence imaging [21] and quantified bone loss using a MATLAB code to measure the total volume of the proximal tibia at the time point identified in the figure [35, 36]. For imaging Akt and ERK activation in the bone marrow, we euthanized mice, immediately used a Dremel tool to think cortical bone of excised tibias, and imaged KTR cells ex vivo by two-photon microscopy as described for lung metastases. We quantified the activation of ERK and Akt in single cells using custom MATLAB code [18].

### Bioinformatics analysis of patient data

We analyzed the expression of PAI1, EPCAM, and PISD in primary CTCs as previously described [37]. For metastasis-free survival plots, we downloaded gene expression data from three different cohorts of patients with breast cancers [38–40] (572 total patients with outcomes) from the National Center for Biotechnology Information (NCBI) Gene Expression Omnibus (GEO) website. We pooled data from GSE2034, GSE2603, and GSE12276 and normalized them together using the Robust Multi-chip Average (RMA) algorithm. We analyzed annotated patient data for survival by Kaplan–Meier analysis. To derive optimal cutoff values, we ordered gene expression levels from low to high and computed a rising step function to define a threshold (middle of the step) by the StepMiner algorithm [41]. We converted gene expression values (PISD: 202392_s_at; MFN1: 211801_x_at; MFN2: 216205_s_at) to high and low levels based on the StepMiner threshold. We estimated time-dependent survival probabilities with the Kaplan–Meier method and compared groups using the log-rank test. We obtained expression data of PISD, Drp1, Mfn1, and Mfn2 at 5 years from initial diagnosis from Oncomine (www.oncomine.org) using the TCGA Breast dataset.

### Statistical analysis

We used a non-parametric Mann–Whitney *U* test for comparisons of cell migration and motility in the microfluidics device. For experiments comparing only two groups, we used a two-tailed, unpaired student’s *t* test. For experiments comparing multiple groups, we used one-way ANOVA and Tukey’s multiple comparisons test. We considered a significance level of *p* < 0.05 statistically significant. We prepared bar graphs (mean values + SD or SEM as denoted in figure legends) and box plots and whiskers using GraphPad Prism 7 or Origin 9.0. For box plots and whiskers, the bottom and top of a box define the first and third quartiles, and the band inside the box marks the second quartile (the median). The ends of the whiskers represent the 10th percentile and the 90th percentiles, respectively. For cell migration in our microfluidics device, the “□” inside the box indicates the mean, dots outside the box and whiskers indicate outliers, and the “x” refers to the maximum and minimum of all data. For all other box plots and whiskers, the “+” within the box refers to the mean. Dots outside the box and whiskers for the clinical data indicate the maximum and minimum values.

## Results

### Phosphatidylserine decarboxylase (PISD) reduces metastasis and osteolytic bone lesions

**Fig. 1:**
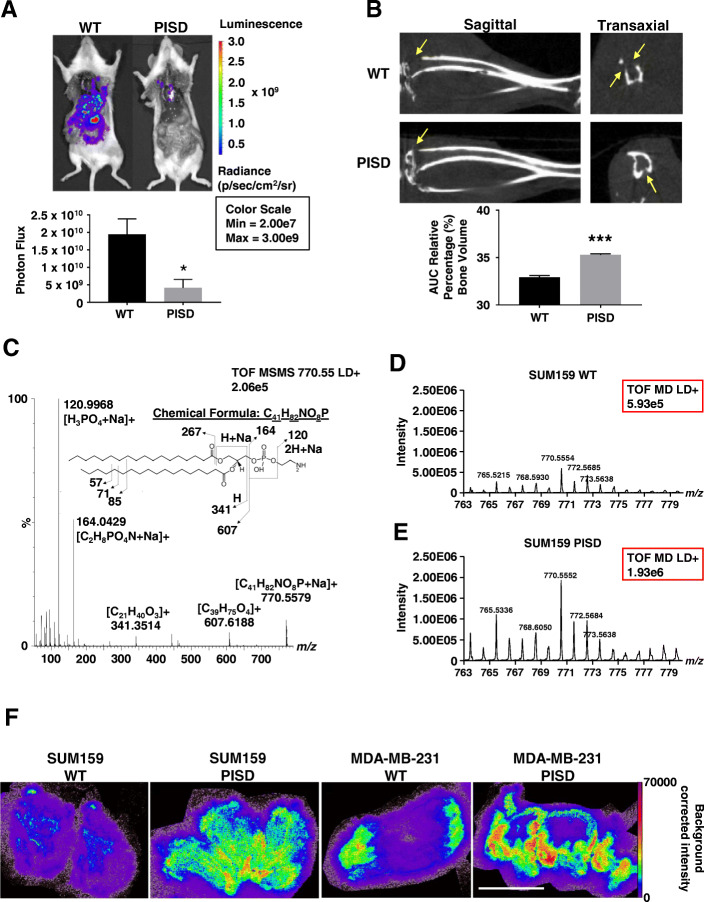
PISD reduces metastasis of triple-negative breast cancer cells. **a** Bioluminescence images of mice injected via the left cardiac ventricle with MDA-MB-231-WT control or PISD expressing cells depict reduced metastatic burden for mice injected with cells that stably express PISD. Graph shows mean whole-body photon flux + SEM for mice that survived to the experimental endpoint (*n* ≥ 7). *p < 0.05. **b** Sagittal and transaxial computed tomography (CT) images of tibias obtained 40 days after femoral artery injection of MDA-MB-231-WT or PISD cells. Images demonstrate reduced osteolytic bone metastases (arrows) in mice with MDA-MB-231-PISD cells. Graph displays mean values for area-under-the-curve for remaining bone volume in proximal tibia + SEM (*n* = 6). ***p < 0.0001. **c** MALDI-MS/MS spectrum of the sodiated ion (770.55 *m/z*) of 18:0 PE from cell pellets. **d**, **e** Graphs show mass spectrometry time-of-flight (TOF) signal intensity (red boxes) of 18:0 PE (770.55 *m*/*z*) in cell pellets from SUM159-WT (**d**) and PISD cells (**e**). **f** MALDI imaging mass spectrometry images of the sodiated ion of 18:0 PE detected at *m/z* 770.552 in orthotopic breast tumors from SUM159- or MDA-MB-231-WT or PISD cells. Scale bar = 5 mm

Using an orthotopic xenograft model, we previously showed that overexpression of phosphatidylserine decarboxylase (PISD) in breast cancer cells significantly reduced local tumor growth [15]. To investigate effects of PISD on metastasis independent of local tumor growth, we used two complementary models that bypass local tumor growth and/or invasion and selectively interrogate tumor extravasation and growth at sites of metastatic seeding. First, we injected MDA-MB-231 human breast cancer cells stably expressing PISD (MDA-MB-231-PISD) or control (WT) breast cancer cells (Figure S1A) directly into the left ventricle of female NSG mice. Breast cancer cells expressed click beetle green (CBG) luciferase for bioluminescence imaging of experimental metastases. Twenty-one days after injection, whole-body bioluminescence imaging revealed significantly reduced total metastatic burden in mice injected with MDA-MB-231-PISD cells (Fig. 1a). Liver, but not lung, metastases occurred in significantly fewer mice injected with MDA-MB-231-PISD cells (Table 1). Additionally, bioluminescence imaging of the flushed bone marrow from the femur and tibia demonstrated a trend toward reduced metastatic burden of MDA-MB-231-PISD cells relative to control (*p* = 0.074, Figure S1B).

**Table 1:** Frequency of mice with metastases from intracardiac injection of MDA-MB-231-wild-type (WT) control and phosphatidylserine decarboxylase (PISD) stably expressing cells

	WT	PISD
Total number of mice	9	7
Number of mice with lung metastases (percentage of mice)	9 (100)	7 (100)
Number of mice with liver metastases (percentage of mice)	9 (100)	4 (57)

As a second experimental model of metastasis, we focused on metastatic disease in the bone, one of the most common sites of breast cancer metastases. We injected MDA-MB-231-PISD or WT cells directly into femoral arteries of mice to selectively produce bone metastases. Computed tomography (CT) of tibias demonstrated less extensive osteolytic lesions in mice injected with MDA-MB-231-PISD cells (Fig. 1b). Together, these data establish that increased levels of PISD reduce the ability of breast cancer cells to metastasize.

### PISD expression increases phosphatidylethanolamine (PE) levels in cells and tumors

PISD converts phosphatidylserine (PS) to phosphatidylethanolamine (PE) in mitochondria, so we expected stable overexpression of this enzyme to elevate amounts of PE in breast cancer cells. MALDI mass spectrometry showed that cultured human SUM159-PISD breast cancer cells contained ~ 3-fold higher levels of PE (770.55 *m/z*, Fig. 1c) than WT cells (Fig. 1d, e). To verify that stable expression of PISD elevates amounts of PE in vivo, we analyzed orthotopic tumor xenografts of PISD and WT cells by MALDI imaging mass spectrometry. Both tumors formed by SUM159- or MDA-MB-231-PISD cells contained ~ 2-fold higher levels of PE than tumors formed from matched WT cells (Fig. 1f and Figure S1C). Elevated levels of PE promote fission of membrane-bound organelles by inducing a large, negative membrane curvature [42], accounting for our prior observation that PISD expression promotes mitochondrial fission [15].

### Mitochondrial fission impacts membrane order

**Fig. 2:**
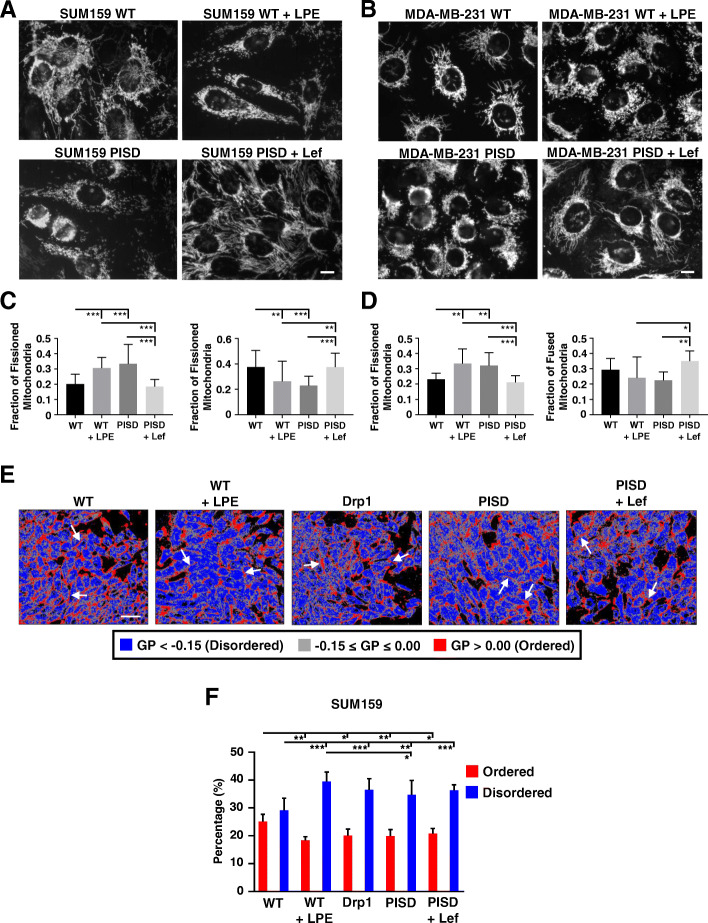
Mitochondrial fission reduces membrane order. **a**, **b** Fluorescence images of SUM159- (**a**) or MDA-MB-231- (**b**) WT and PISD cells stably expressing mitochondrially targeted GFP (Mito-GFP) treated with vehicle control, α-lysophosphatidylethanolamine (LPE, 50 μM), or leflunomide (Lef, 50 μM) for 48 h. Scale bar = 10 μm. **c**, **d** Graphs show the mean fraction + SD of fissioned or fused mitochondria after treatment with vehicle control, LPE, or Lef (*n* ≥ 13 images for each treatment group) in SUM159 (**c**) or MDA-MB-231 (**d**) cells. *p < 0.05. **p < 0.01. ***p < 0.0001. **e** Representative images of SUM159-WT, WT treated with α-lysophosphatidylethanolamine (LPE, 50 μM), PISD, PISD treated with leflunomide (Lef, 50 μM), or Drp1 cells stained with laurdan. Images show pseudocolored general polarization (GP) values. Red color indicates areas of high membrane order and less dynamics, whereas blue color denotes areas of low membrane order and more dynamics. Gray color indicates areas that fell between the ordered and disordered phases based upon an arbitrary cutoff used for all groups. Arrows point to cell membranes. Scale bar = 50 μm. **f** Graph shows mean + SD for the percentage of all ordered and disordered pixels for each image in **e** (*n* = 10 images per group). *p < 0.05. **p < 0.01. ***p < 0.0001

Our previous work suggests that PISD inhibits tumor growth and metastasis by inducing mitochondrial fission. To further explore this hypothesis, we next used compounds to enforce mitochondrial fission and fusion in cells. Since PISD expression increases levels of PE in cells, we treated cells with l-α-lysophosphatidylethanolamine (LPE), a cell permeant analog of the PISD enzymatic product that cells efficiently convert to PE within mitochondria [43, 44]. Using fluorescence microscopy, we found that treatment of WT cells with LPE drove mitochondrial fission with a similar phenotype to PISD cells (Fig. 2a–d). Conversely, we treated cells with leflunomide (Lef), an FDA-approved drug recently validated to stimulate expression of the mitochondrial fusion genes, mitofusins 1 and 2 (Mfn1/2) [45, 46], to promote mitochondrial fusion. Consistent with previous literature [45], leflunomide promoted mitochondrial fusion in PISD cells (Fig. 2a–d).

We next identified effects of LPE and leflunomide on functions of mitochondria. Mitochondrial fission induced by LPE slightly reduced mitochondrial mass, while fusion driven by treatment with leflunomide increased mitochondrial mass as assayed by MitoTracker Green (Figure S2A). WT cells treated with LPE (mitochondrial fission) exhibited lower mitochondrial membrane potential (Figure S2B), while leflunomide increased mitochondrial membrane potential in PISD cells (Figure S2B). We did not detect significant changes in cell size between WT, PISD, and cells treated with LPE or leflunomide (Figure S2C). Although mitochondrial morphology and membrane potential regulate oxidative phosphorylation, levels of ATP did not differ between WT and PISD cells for both SUM159 (Figure S2D) and MDA-MB-231 (Figure S2E) cells.

In addition to promoting mitochondrial fission, stable expression of PISD increased levels of PE in cells and tumors. Since phosphatidylethanolamine typically reduces ordered domains in cell membranes, we used a validated fluorescent probe of membrane polarity, laurdan [31], to quantify overall effects on degree of lipid packing, thickness of the lipid bilayer, and the rotational freedom of lipids within the bilayer. We found that expression of PISD significantly reduced plasma membrane order in both SUM159 (Fig. 2e, f) and MDA-MB-231 cells (Figure S3A-B). Consistent with the expression of PISD, we found that treating cells with LPE reduced membrane order and promoted membrane disorder in SUM159 (Fig. 2e, f) and MDA-MB-231 (Figure S3A-B) cells. Treatment of PISD cells with leflunomide did not change membrane order in either cell line. As a complimentary approach to drive mitochondrial fission, we stably expressed Drp1 in cells (Figure S4A-B) [19]. Stable expression of Drp1 promoted mitochondrial fission (Figure S4C-F). Consistent with LPE and PISD, stably expressing Drp1 reduced membrane order and promoted membrane disorder in SUM159 (Fig. 2e, f) and MDA-MB-231 (Figure S3A-B) cells. Together, these results demonstrate that lipid changes and mitochondrial phenotype directly impact membrane order in cells. More specifically, mitochondrial fission promotes membrane disorder.

### Reduced Akt and ERK signaling in cells with fissioned mitochondria

**Fig. 3:**
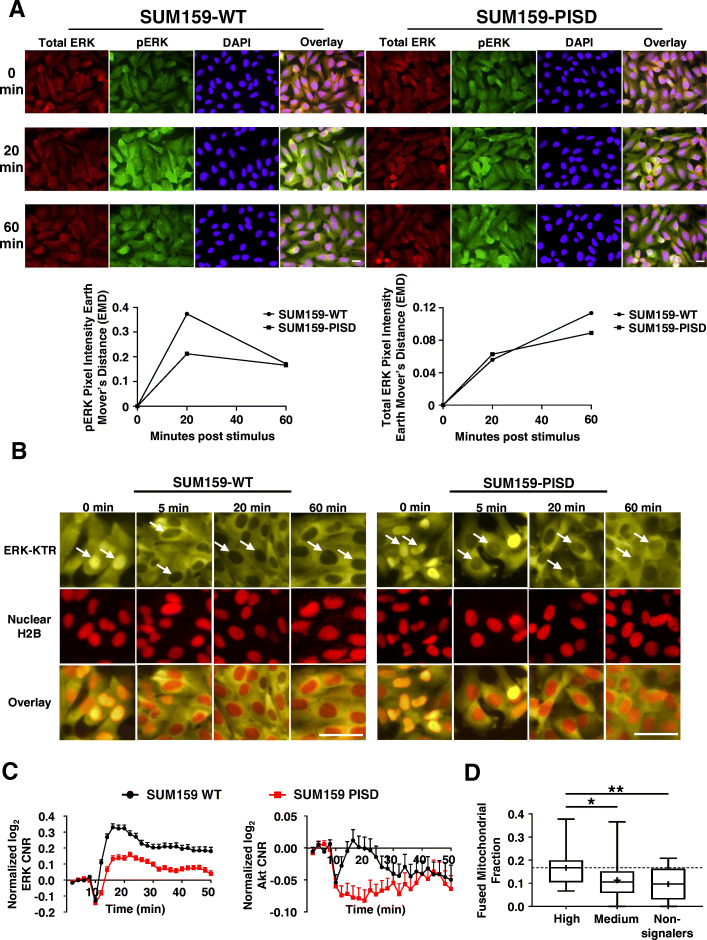
Elevated expression of PISD reduces signaling to ERK. **a** Representative overlaid images of immunofluorescence staining of total (red) ERK, phosphorylated (green) ERK, and DAPI (nuclei, blue) in SUM159-WT and PISD cells at the initial time point (0 min) and 20 and 60 min after addition of serum (10%). PISD cells exhibit reduced amplitude and duration of phosphorylated ERK (decreased green in the overlaid images at 20- and 60-min time points). Scale bar is 20 μm. Bottom: Graphs show earth mover’s distance (EMD) of the green intensity (pERK, left) or red intensity (total ERK, right) between each time point after addition of stimulus (10% serum) relative to the initial time point for Akt (*n* = 5 images per group). **b** Fluorescence images of ERK KTR (yellow) and nuclear H2B-mCherry (red) in SUM159-WT and PISD cells at the initial time point (0 min) and 5, 20, and 60 min after addition of 10% serum show translocation of yellow fluorescence out of the nucleus (arrows, darker nucleus = ERK activation). Scale bar = 50 μm. **c** Graphs show mean + SEM for activation of Akt and ERK in response to serum, expressed as log_2_ of cytoplasmic/nuclear fluorescence intensities normalized to time 0 for an average cell in each cell type. **d** Box plot and whiskers for the fraction of fused mitochondria in high, medium, or non-signaling SUM159-WT cells demonstrates enhanced signaling in response to serum in cells with a higher fraction of fused mitochondria. Line within the box denotes the median, and the “+” symbol denotes the mean. Dashed line represents the median fused fraction in high signaling cells (*n* = 96). *p < 0.05. **p < 0.01

Previous work demonstrates that dynamic changes in signaling inputs to mitochondria regulate mitochondrial morphology [47–49]. Only limited information exists about reciprocal regulation between morphological heterogeneity of mitochondria and effects on cancer cell signaling. Furthermore, areas of increased membrane order, called lipid rafts, facilitate receptor activation and downstream signaling [50]. Since cells with greater mitochondrial fission show reduced order of membranes, we investigated to what extent enforcing changes in mitochondrial morphology regulated activities of Akt and ERK, two kinases in signaling pathways mutated in over 30% of all cancers [51]. Due to mutations in PI3K in SUM159 cells and KRas in MDA-MB-231 cells causing constitutive activation of Akt and ERK, respectively, we focused most assays on kinase activities of ERK in SUM159 and Akt in MDA-MB-231 cells, respectively. First, to identify effects of PISD on levels of Akt and ERK, we performed immunofluorescence staining for phosphorylated (activated) and total ERK in SUM159 and Akt in MDA-MB-231 cells, respectively, following treatment with serum. Immunostaining showed reduced activation of ERK (Fig. 3a, S5A-B) and Akt (Figure S5C-E) in cells stably expressing PISD compared to WT cells.

To identify more dynamic effects on signaling, we used breast cancer cells stably expressing fluorescent kinase translocation reporters (KTRs) for Akt and ERK. The KTR reporters utilize known downstream substrates specific for Akt or ERK. Phosphorylation of the substrate drives a reversible translocation of the reporter into and out of the nucleus (Figure S6A) [27, 28]. KTRs permit quantitative imaging studies of Akt and ERK kinase activities in single cells or populations. Since our KTR construct expresses H2B-mCherry to denote the nucleus, we performed remaining experiments with cells stably expressing a non-fluorescent PISD. qRT-PCR analysis revealed higher expression of PISD in stably transduced SUM159- and MDA-MB-231-PISD cells compared to WT cells (Figure S1D).

Using cells stably expressing KTRs for Akt and ERK, we found that PISD reduced both amplitude and duration of activation of both Akt and ERK in response to serum, as quantified by changes in localization of imaging reporters from nucleus (kinase “off”) to cytoplasm (kinase “on”) (Fig. 3b, c and S6B-C). While we designate “off” and “on” locations for KTRs, we emphasize these reporters give analog, not digital, measurements of kinase activity.

To further analyze effects of mitochondrial fission on cell signaling, we next subdivided cells into high, medium, and non-signalers based upon total area-under-the-curve for signaling. We found that the expression of PISD shifted cells to the non-signaling group in SUM159 (Figure S7A) and MDA-MB-231 (Figure S6D) cells, further demonstrating that mitochondrial fission inhibited cell signaling. Comparing average signaling curves over time for ERK (Figure S7A) and Akt (Figure S6D) in high, medium, and non-signalers, we found reduced peak amplitude of signaling in most groups in PISD cells.

To directly correlate mitochondrial morphology with signaling output, we analyzed mitochondrial morphology prior to addition of serum and kinase activities stimulated by serum in single WT cells. WT cells with the highest fraction of fused mitochondria showed significantly greater activation of ERK (Fig. 3d) and Akt (Figure S6E). Conversely, cells with greater fractions of fissioned mitochondria exhibited reduced activities of both kinases (Figure S7B-C). Together, these data show that mitochondrial fission correlates with reduced activation of Akt and ERK, while mitochondrial fusion promotes signaling by these kinases.

**Fig. 4:**
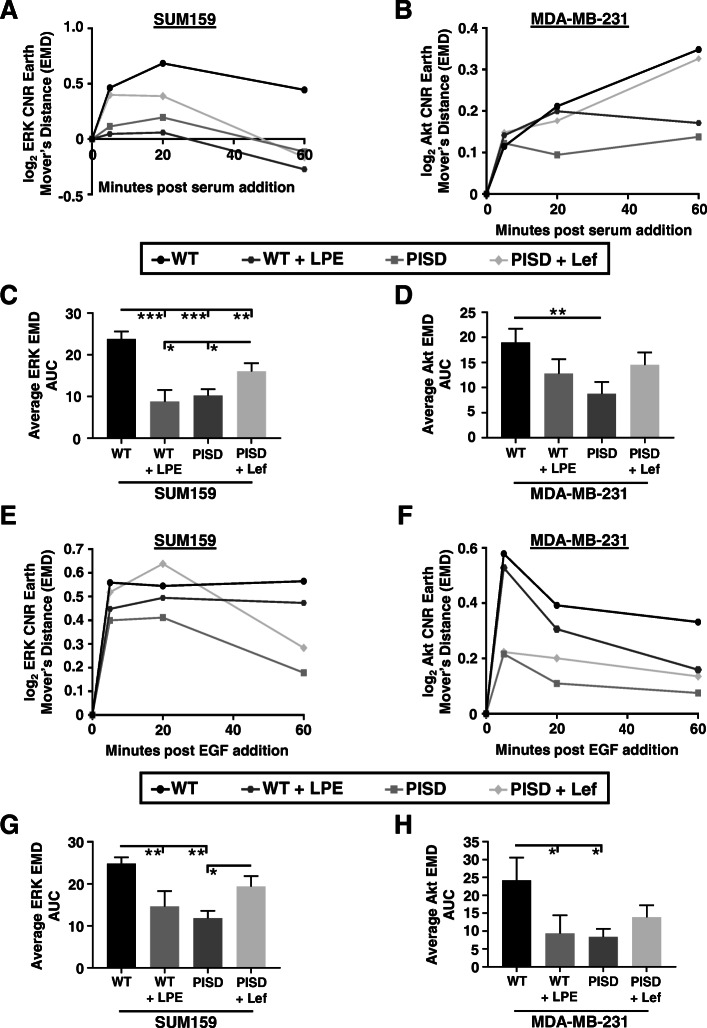
Fused mitochondria potentiate ERK and Akt signaling in response to different stimuli. We quantified activation of Akt and ERK in SUM159 and MDA-MB-231 cells, respectively, by imaging KTRs. Graphs show earth mover’s distance (EMD) and area-under-the-curve (AUC) for 0, 5, 20, and 60 min after addition of stimulus (10% serum (**a**–**d**) or EGF (50 ng/mL, **e**–**h**)) relative to baseline signaling before stimulus (0 min) (*n* ≥ 188 cells per group)

To further analyze effects of mitochondrial morphology on signaling through Akt and ERK, we enforced mitochondrial fission and fusion with LPE and leflunomide, respectively, and then measured signaling in response to serum. Consistent with results comparing PISD and WT cells, we found that increasing mitochondrial fission with LPE blunted activation of Akt and ERK in WT cells, while driving fusion with leflunomide potentiated signaling (Fig. 4a–d). We observed similar results in cells stimulated with epidermal growth factor (EGF). Again, mitochondrial fission driven by expression of PISD or treatment with LPE blunted ERK and Akt signaling in response to EGF (Fig. 4e–h). Cells with a greater extent of fused mitochondria responded to EGF with enhanced activation of Akt and ERK. We also tested effects of stably expressed Drp1 in both SUM159 and MDA-MB-231 cells. Consistent with PISD and WT cells treated with LPE, stable expression of Drp1 also reduced signaling to Akt and ERK in response to serum (Figure S8A-D) and EGF (Figure S8E-H). Collectively, these data show that mitochondrial fragmentation inhibits ERK and Akt signaling in response to various stimuli.

### Interventions driving mitochondrial fission inhibit ERK and Akt signaling in metastases

Because mitochondrial fission results in reduced signaling, we hypothesized that mitochondrial fission versus fusion phenotypes function as general regulators of processes driving metastasis, such as migration and invasion. We treated breast cancer cells with LPE or leflunomide and then quantified two-dimensional migration toward serum in a microfluidics device [15]. Consistent with our previous results, cells stably expressing PISD migrated less than corresponding WT cells (Figure S9A-B). We also found that WT cells treated with LPE migrated less than control WT cells, while treating PISD cells with leflunomide increased migration (Figure S9A-B). To identify effects of mitochondrial morphology on migration and invasion in a more complex 3D environment, we next embedded co-culture spheroids of WT or PISD breast cancer cells with human mammary fibroblasts (HMFs) into fibrin gels and treated cells with LPE, leflunomide, or vehicle control. Consistent with migration in our microfluidic device, we found that spheroids with PISD cells displayed a smaller diameter (Figure S9C-D), suggesting PISD reduces cell migration and invasion. Protrusions from the core of spheroids with PISD cells did not extend as far as spheroids from WT cells (Figure S9C and S9E). Furthermore, treating spheroids from WT cells with LPE reduced sphere diameter and invasion into the surrounding extracellular matrix (ECM). Enforcing mitochondrial fusion with leflunomide in spheroids with PISD cells rescued inhibitory effects on spheroid size and invasion (Figure S9C-E). We also observed that stable expression of PISD reduced overall movement of breast cancer cells on tissue-engineered constructs with aligned fibrils of fibronectin (Figure S9F-G). Overall, these data show that mitochondrial morphology dictates cell migration and invasion.

**Fig. 5:**
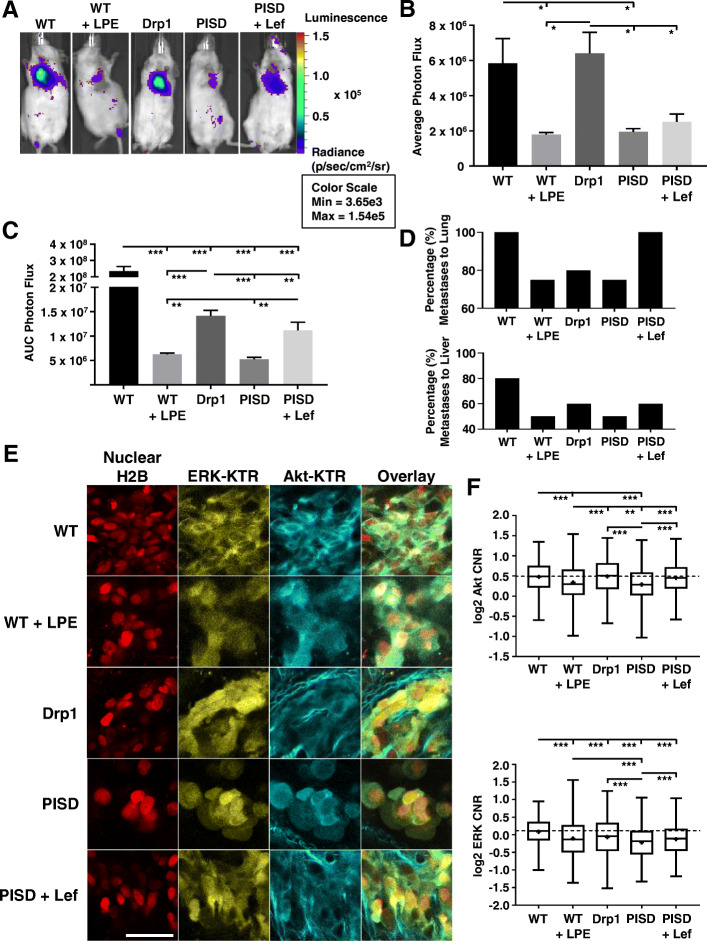
Mitochondrial fission inhibits systemic metastases and oncogenic signaling in vivo. **a** Bioluminescence images of mice 1 day after intracardiac injection with SUM159-WT, WT treated with α-lysophosphatidylethanolamine (LPE, 50 μM), PISD, PISD treated with leflunomide (Lef, 50 μM), or Drp1 cells. **b** Graph shows mean + SEM photon flux values 1 day after injection (*n* ≥ 4 mice per group). *p < 0.05. **c** Mean + SEM for area-under-the-curve (AUC) analysis of photon flux for metastases over 20 days for SUM159 cells described in panel **a**. **p < 0.01. ***p < 0.0001. **d** Graphs display percentages of mice in each group (*n* ≥ 4 mice per group) with metastases to the lung (top) and liver (bottom). **e** Ex vivo fluorescence images of ERK KTR (yellow), Akt KTR, (cyan), and nuclear H2B-mCherry (red) in lung metastatic cells from intracardiac injection with SUM159-WT, WT + LPE, PISD, PISD + Lef, and Drp1 cells. Scale bar = 50 μm. Second harmonic signal shows collagen fibers (cyan) surrounding cells. **f** Box plot and whiskers for quantified log_2_ cytoplasmic/nuclear fluorescence intensities for Akt (top) and ERK (bottom) activities based on the ex vivo imaging of KTRs in lung metastases from each group (*n* ≥ 412 cells per group) in **e**. Line within the box denotes the median, and the “+” symbol denotes the mean. Dashed line represents the median of WT cells. **p < 0.01, ***p < 0.0001. Data demonstrate reduced activation of Akt and ERK from interventions that increase mitochondrial fission prior to injection (LPE treatment of WT cells) or stably throughout the experiment (PISD and Drp1)

To directly assess effects of mitochondrial morphology on metastasis, we injected SUM159-WT, WT treated with LPE, PISD, PISD treated with leflunomide, and Drp1 cells expressing CBG luciferase directly into the left ventricle of female NSG mice. We only treated cells with LPE or leflunomide ex vivo and did not administer these agents to mice after injection of breast cancer cells. One day after injection, whole-body bioluminescence imaging showed that mitochondrial fission induced by PISD or treatment with LPE significantly reduced signal in lungs (Fig. 5a, b). Furthermore, ex vivo treatment of cancer cells with leflunomide modestly increased signal in the lung for PISD cells 1 day after injection (Fig. 5a, b). As measured by area-under-the-curve analysis of bioluminescence over 20 days post-injection, interventions that produced mitochondrial fission prior to injection (LPE treatment of WT cells) and stably throughout the experiment (PISD and Drp1) reduced overall bioluminescence signal compared to WT cells (Fig. 5c). Although WT and Drp1 cells did not differ 1 day after injection (Fig. 5a, b), stable expression of Drp1 reduced subsequent growth of breast cancer cells (Fig. 5c). Additionally, PISD cells treated with leflunomide ex vivo to drive mitochondrial fusion produced significantly higher metastases than matched PISD cells (Fig. 5c). Quantification of organ-specific metastases per group revealed that mice injected with cells with a fissioned morphology showed reduced metastases in the lung and liver (Fig. 5d). Using leflunomide to drive mitochondrial fusion in PISD cells overcame inhibitory effects of PISD on metastases to the lung and liver (Fig. 5d).

We measured Akt and ERK signaling in single cells by two-photon microscopy of Akt and ERK KTRs in SUM159 lung metastases. Shifting mitochondrial morphology through pharmacologic approaches prior to injection (WT cells treated with LPE) or stable gene expression (PISD, Drp1) significantly reduced ERK signaling (Fig. 5e, f). PISD cells and WT cells treated with LPE also showed reduced activity of Akt in lung metastases. As compared with signaling in PISD cells, ex vivo treatment with leflunomide resulted in greater activities of Akt and ERK. Imaging studies of the bone marrow also revealed lower Akt and ERK signaling in metastatic PISD cells in the femur and tibia following femoral artery injection (Figure S10A-C). Overall, these results demonstrate that mitochondrial fission inhibits metastatic potential by limiting Akt and ERK signaling.

### Genes that control mitochondrial morphology correlate with patient prognosis

**Fig. 6:**
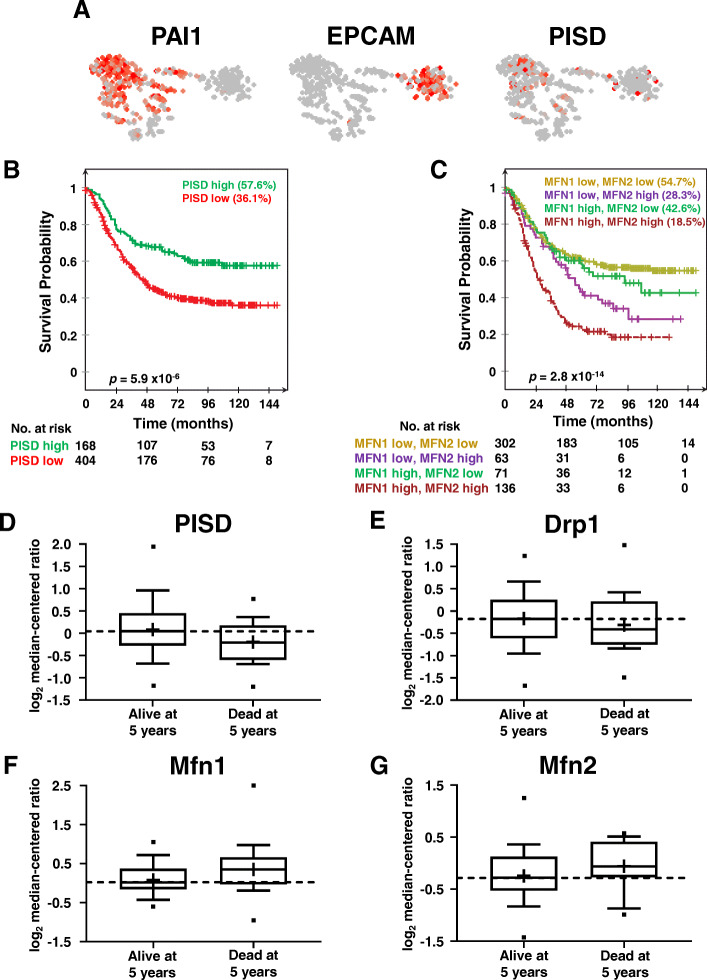
Genes associated with mitochondrial fission correlate with better overall prognosis in breast cancer. **a** Gene expression and clustering of high (red) and low (gray) levels of PAI1, EPCAM, and PISD in primary circulating tumor cells (CTCs) from patients with metastatic breast cancer. Each dot represents a single CTC (*n* = 666 CTCs from 21 breast cancer patients). PAI1, a gene known to correlate with poor prognosis in breast cancer, defines a cluster associated with epithelial-to-mesenchymal transition (EMT), and EPCAM defines the right cluster containing genes previously associated with mesenchymal-to-epithelial transition (MET). **b**, **c** Kaplan–Meier curves for metastasis-free survival over time among patients whose primary tumors had high versus low levels of PISD (**b**) or Mfn1 and Mfn2 (**c**). Percentage indicates metastasis-free survival at endpoint, and *p* values represent significant differences in outcome between all groups displayed on the graph. **d**–**g** Box plot and whiskers for gene expression of PISD (**d**) and Drp1 (**e**) (mitochondrial fission), and MFN1 (**f**) and MFN2 (**g**) (mitochondrial fusion) in patients that were either dead or alive at 5 years after the initial pathological diagnosis (alive at 5 years: *n* = 58, dead at 5 years: *n* = 21 from The Cancer Genome Atlas (TCGA)). Line within the box denotes the median, and the “+” symbol denotes the mean. Dashed line represents median expression of the gene of interest in patients alive at 5 years

Since mitochondrial fission inhibits metastasis and kinase signaling by Akt and ERK, we hypothesized that the expression levels of mitochondrial morphology genes correlate with patient prognosis. We first analyzed the expression of PISD in primary circulating tumor cells (CTCs) from patients with advanced breast cancer. Compared to the expression of PAI1, a gene known to correlate with worse outcome in patients [17], or EPCAM, a gene that is commonly overexpressed in breast cancer, breast CTCs expressed low levels of PISD (Fig. 6a). Survival analysis revealed that low PISD expression correlated significantly with poorer metastasis-free survival (MFS, Fig. 6b). We also found that high expression of mitofusins (Mfn1/2) correlated with poorer MFS to the bone and brain in patients with breast cancer (Fig. 6c). High levels of both Mfn1 and Mfn2 synergistically reduced MFS, suggesting interactions among these drivers of mitochondrial fusion in disease progression. Data from the TCGA for breast cancer demonstrated higher levels of PISD (Fig. 6d) and Drp1 (Fig. 6e) in patients alive 5 years after initial diagnosis. Patients alive 5 years after presentation also express lower levels of mitochondrial fusion genes Mfn1 (Fig. 6f) and Mfn2 (Fig. 6g). In summary, high levels of mitochondrial fission genes correlate with better prognosis for patients with breast cancer.

## Discussion

In this study, we provide the first evidence to our knowledge that mitochondrial fission inhibits metastasis in triple-negative breast cancer. Gene expression data from patients reveals that genes associated with mitochondrial fission correlate with better overall survival, while fusion genes correlate with worse overall survival. Using in vitro and in vivo studies, we demonstrate that enforcing mitochondrial fission not only inhibits migration and invasion of breast cancer cells but also blunts signaling through ERK and Akt. Moreover, enforcing mitochondrial fusion can reverse many inhibitory effects of mitochondrial fission. These results highlight mitochondrial dynamics as a central node in tumor progression that connects external stimuli to downstream signaling events.

Building on our previous discovery that PISD drives mitochondrial fission and inhibits breast tumor growth [15], we establish that PISD also inhibits metastasis and bone destruction. Here, we show that increased PE, either by PISD expression or exogenous l-α-lysophosphatidylethanolamine (LPE), drives mitochondrial fission. While recognizing that exogenous addition of LPE may have off-target effects, treatment of cells with LPE recapitulated many phenotypes associated with PISD-driven mitochondrial fission. In cells, PE functions as a structural component of membranes where it regulates membrane fission and fusion events [52, 53]. Thus, mitochondrial fission likely occurs due to an increase in negative membrane curvature induced by higher incorporation of PE in the mitochondrial membrane [42]. We reversed this phenotype by treating cells with leflunomide, demonstrating that induction of mitofusins (Mfn1/2) overcomes PISD-induced mitochondrial fission.

This study reveals that mitochondrial fission reduces order of cell membranes, providing a potential mechanism for effects on cell signaling. Lipid composition of cell membranes regulates receptors and other signaling molecules. In particular, areas of high membrane order, known as lipid rafts, facilitate receptor activation and signaling cascades [50]. Disrupting ordered domains in the plasma membrane impairs activation of major families of cell surface receptors, including receptor tyrosine kinases such as EGFR and G-protein coupled receptors [54, 55]. Since PE made by PISD in mitochondria can traffic to the plasma membrane [56], either overexpressing this enzyme or treatment with LPE may increase local amounts of this non-raft-forming lipid and reduce magnitude of signaling from multiple inputs. More broadly, recent studies demonstrate that proteins that drive mitochondrial fission (Drp1) and fusion (Mfn1/2) regulate the abundance of PE, other lipid species, and lipid saturation in cells [57–59]. Therefore, effects of mitochondrial phenotypes on lipid composition suggest a common mechanism through which mitochondrial phenotypes regulate membrane order and signal transduction in cancer [32].

Prior studies report discordant effects of mitochondrial morphology in cancer. Initial research demonstrated increased mitochondrial fission in invasive cancers, correlating with enhanced metastatic potential [47, 60]. Therefore, inhibiting mitochondrial fission or promoting mitochondrial fusion suppressed malignant phenotypes [46, 61]. However, recent work contradicted these results, demonstrating mitochondrial fusion as a driver of epithelial-mesenchymal transition (EMT) [62], enhanced cell migration [63, 64], and initiation and maintenance of a stem cell phenotype [65, 66]. Mitochondrial fusion also correlated with enhanced numbers of primary circulating tumor cells (CTCs) and metastasis [67]. Our current study demonstrates that mitochondrial fission limits breast cancer migration, invasion, and metastasis. Additionally, we show that enforcing mitochondrial fusion with leflunomide can overcome inhibitory effects of PISD to drive fission. These results support pro-oncogenic functions of mitochondrial fusion. Discrepancies between outcomes produced by mitochondrial fission versus fusion may arise from different inherent oncogenic driver mutations in cancer cells used for these studies. For example, MAPK-driven oncogenesis induced mitochondrial fission [47, 68, 69], while MYC-driven cancer promoted a fused morphology [70, 71]. As a complex, multi-step process requiring many mitochondrial-dependent programs, mitochondrial fission may promote or inhibit metastasis depending not only on genetic, but also environmental and tissue differences among tumors [1]. Defining contexts in which different mitochondrial morphologies favor tumor progression warrants further investigation in vitro and in vivo.

PI3K/Akt/mTOR and Ras/Raf/MEK/ERK pathways are two of the most commonly dysregulated signaling pathways in TNBC and other cancers [72, 73]. Our data show that mitochondrial fission blunts ligand-dependent activation of Akt and ERK. Previous work demonstrated localization of Akt to mitochondria [11, 12], and association with mitochondria-associated membranes (MAMs) leads to further activation by mTORC2 [74, 75]. Since formation of MAMs depends on mitofusin homo- or heterodimerization [76–78], we suggest that enforcing mitochondrial fission reduces the formation of MAMs and activation of Akt. While few studies have addressed physiological importance of ERK localization to mitochondria in cancer, association of ERK with mitochondria promotes dimer formation [79], an essential step in signaling by these kinases. Complete activation of ERK requires mitochondria [80], suggesting that mitochondrial proteins serve as scaffolds to assemble dimers of ERK and control amplitude and duration of signaling [81]. Since membrane structure and composition regulate functions of scaffold proteins [82, 83], our data suggest that shifts to fission interfere with scaffolding of ERK on mitochondria to limit ERK signaling. We recognize that other pathways such as mitochondrial membrane potential and reactive oxygen species (ROS) may also contribute to altered signaling. Therefore, more work is needed to identify mechanisms underlying altered mitochondrial morphology and PI3K/Akt/mTOR and Ras/Raf/MEK/ERK signaling pathways.

We found that mitochondrial fission reduces lung colonization and growth in a mouse model of breast cancer. Furthermore, our data show that either transient or stable induction of mitochondrial fission reduces ERK and Akt signaling in lung metastases. Evidence suggests that changes in mitochondrial metabolism lead to an accumulation of key intermediates, such as S-adenosylmethionine (SAM), necessary to regulate gene expression [14, 84]. We speculate that transient or stable changes to mitochondrial morphology alter specific metabolites that play either a direct or indirect role in regulating epigenetic mechanisms that persist to reduce both metastasis and cellular signaling. Mitochondrial fission/fusion dynamics and metabolism are highly linked and regulate each other. For example, transient or stable loss of Drp1 not only drives mitochondrial fusion but also causes global metabolic reprogramming away from glycolysis. More specifically, Drp1 loss alters phospholipid metabolism [57, 58], such as phosphatidylserine (PS), directly linking mitochondrial dynamics and phospholipid metabolism. Moreover, phosphatidylethanolamine (PE) functions as an important SAM methylation acceptor and regulates phosphorylation-based signal transduction [85]. Therefore, our data suggest that enforcing mitochondrial phenotypes may have lasting epigenetic consequences, likely through changes in cellular phospholipids, that regulate breast cancer signaling and metastasis.

Our data point to mitochondrial transitions between fission and fused states as a central regulatory hub for tumor initiation and metastasis in breast cancer [1, 2]. By limiting signaling through Akt and ERK, mitochondrial fission diminishes activation of two major drivers of breast cancer and multiple other malignancies. Mitochondrial fission typically reduces efficiency of oxidative phosphorylation, a metabolic process upregulated in metastatic breast cancer cells [86, 87] and breast CSCs [88]. Therefore, strategies to shift mitochondria from fused to fissioned states may block key processes needed for tumor progression. Recent work demonstrates that ingestion of stearic acid, a saturated lipid, rapidly stimulates mitochondrial fusion in human subjects [89]. Potentially, administration of LPE could provide a metabolite-based approach to drive mitochondrial fission. Overall, this study points to mitochondrial dynamics as a new target for therapy in breast cancer, motivating the development of pharmacologic agents and other treatment approaches to drive cells toward mitochondrial fission.

## Conclusions

In this study, we determined for the first time that mitochondrial fission inhibits triple-negative breast cancer (TNBC) progression and metastasis by reducing migration, invasion, and signaling through Akt and ERK. These data suggest that therapies driving mitochondrial fission may benefit patients with breast cancer.

## Supplementary information

**Additional file 1: **Supplemental **Figure S1** PISD increases PE levels in tumors and promotes mitochondrial fission. Supplemental **Figure S2**. Enforcing mitochondrial phenotypes alters mitochondrial mass and membrane potential. Supplemental **Figure S3**. Mitochondrial fission increases membrane disorder in MDA-MB-231 cells. Supplemental **Figure S4**. Drp1 drives mitochondrial fission. Supplemental **Figure S5**. PISD reduces ERK and Akt phosphorylation. Supplemental **Figure S6**. Fissioned mitochondria reduce signaling output to Akt in single cells. Supplemental **Figure S7**. PISD reduces the number of high signalers in SUM159 cells. Supplemental **Figure S8**. Drp1 reduces ERK and Akt signaling. Supplemental **Figure S9**. Mitochondrial fission reduces cell migration. Supplemental **Figure S10**. PISD reduces oncogenic signaling in the bone marrow niche.

## Data Availability

We will provide data generated or methods used to analyze data upon request.

## Abbreviations

ATP: Adenosine triphosphate
CBG: Click beetle green
CSCs: Cancer stem cells
CT: Computed tomography
CTCs: Circulating tumor cells
Drp1: Dynamin-related protein 1
EGF: Epidermal growth factor
EMD: Earth mover’s distance
EPCAM: Epithelial cell adhesion molecule
ERK: Extracellular regulated kinase
GP: General polarization
KTR: Kinase translocation reporter
Lef: Leflunomide
LPE: l-α-Lysophosphatidylethanolamine
MALDI: Matrix-assisted laser desorption/ionization
Mfn1/2: Mitofusin 1/2
MFS: Metastasis-free survival
NSG: NOD *scid* gamma
Opa1: Optic atrophy 1
PAI1: Plasminogen activator inhibitor 1
PISD: Phosphatidylserine decarboxylase
PE: Phosphatidylethanolamine
PS: Phosphatidylserine
ROS: Reactive oxygen species
TCGA: The Cancer Genome Atlas
TNBC: Triple-negative breast cancer
