# Supplementary material for: Enhanced mitochondrial fission suppresses signaling and metastasis in triple-negative breast cancer
Source: Breast Cancer Res. 2020 Jun 5;22:60. doi: 10.1186/s13058-020-01301-x (PMC7275541; doi:10.1186/s13058-020-01301-x)
Supplement: Supplementary file 1 — Additional file 1: Supplemental Figure S1 PISD increases PE levels in tumors and promotes mitochondrial fission. Supplemental Figure S2. Enforcing mitochondrial phenotypes alters mitochondrial mass and membrane potential. Supplemental Figure S3. Mitochondrial fission increases membrane disorder in MDA-MB-231 cells. Supplemental Figure S4. Drp1 drives mitochondrial fission. Supplemental Figure S5. PISD reduces ERK and Akt phosphorylation. Supplemental Figure S6. Fissioned mitochondria reduce signaling output to Akt in single cells. Supplemental Figure S7. PISD reduces the number of high signalers in SUM159 cells. Supplemental Figure S8. Drp1 reduces ERK and Akt signaling. Supplemental Figure S9. Mitochondrial fission reduces cell migration. Supplemental Figure S10. PISD reduces oncogenic signaling in the bone marrow niche. [file 13058_2020_1301_MOESM1_ESM.pdf]

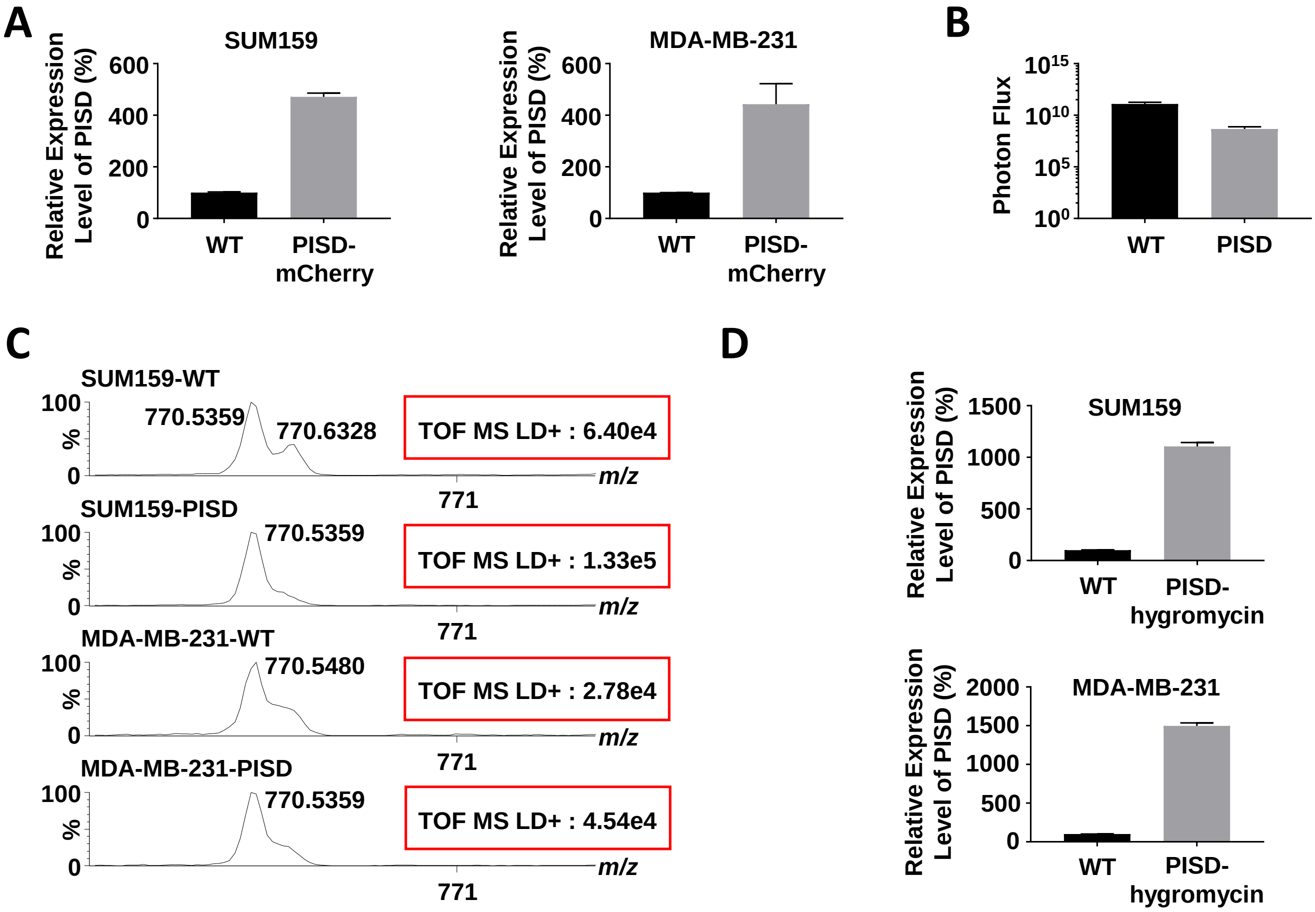

Supplemental Figure S1. PISD increases PE levels in tumors and promotes mitochondrial fission. A. qRT-PCR results demonstrate > 3-fold overexpression of PISD in both SUM159 and MDA-MB-231 triple negative breast cancer cells. Graph shows mean values + SD expression relative to WT cells (n = 3). **B.** Graph shows mean values + SEM for photon flux from bone marrow collected 20 days after intracardiac injection of MDA-MB-231-WT (n = 9 mice) or PISD stably expressing cells (n = 7 mice). **C.** MALDI-imaging mass spectrometry of the 770.5552 peak showing the intensity of sodiated 18:0 PE in tumors formed from SUM159- and MDA-MB-231-WT and PISD stably expressing cells. **D.** qRT-PCR results demonstrate that PISD is overexpressed in both SUM159- and MDA-MB-231-PISD-hygromycin cells relative to WT cells. Graph shows mean values + SD expression relative to WT cells (n = 3).

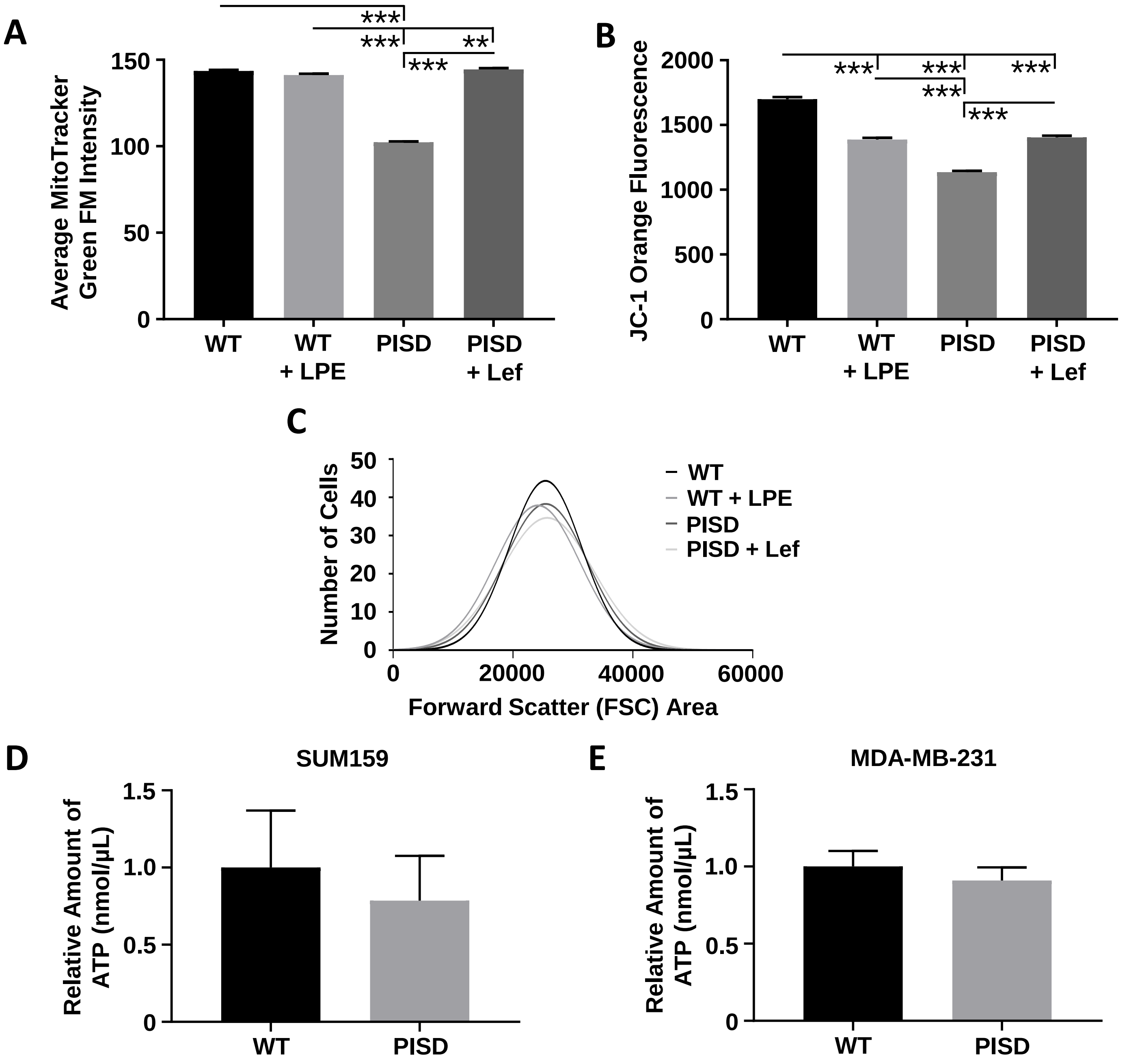

Supplemental Figure S2. Enforcing mitochondrial phenotypes alters mitochondrial mass and membrane potential. A. Graphs show flow cytometry data from SUM159-WT cells treated with α-lysophosphatidylethanolamine (LPE) or vehicle, or SUM159-PISD stably expressing cells treated with leflunomide (Lef) or vehicle before staining with MitoTracker Green for flow cytometry. Graphs show mean + SEM (n ≥ 10,003 cells per group). Higher MitoTracker Green staining indicates increased mass of mitochondria per cell. ** = $p < 0.01$. *** = $p < 0.0001$. **B.** Graphs show flow cytometry data for SUM159-WT cells treated with LPE or vehicle, or SUM159-PISD stably expressing cells treated with Lef or vehicle before staining with JC-1 for flow cytometry. Graphs show mean orange intensity + SEM (n ≥ 7523 cells per group). A reduction in orange intensity refers to reduced mitochondrial membrane potential. *** = $p < 0.0001$. **C.** Histogram shows flow cytometry data for forward scatter area in SUM159-WT, WT treated with LPE, PISD and PISD treated with leflunomide (n ≥ 10,000 cells per group). Data show no change in forward scatter area (cell size) after treatment with LPE or leflunomide. **D, E.** Normalized ATP measurements in SUM159- (left) and MDA-MB-231- (right) WT and PISD cells. Graphs show mean values + SD relative to WT cells (n ≥ 3).

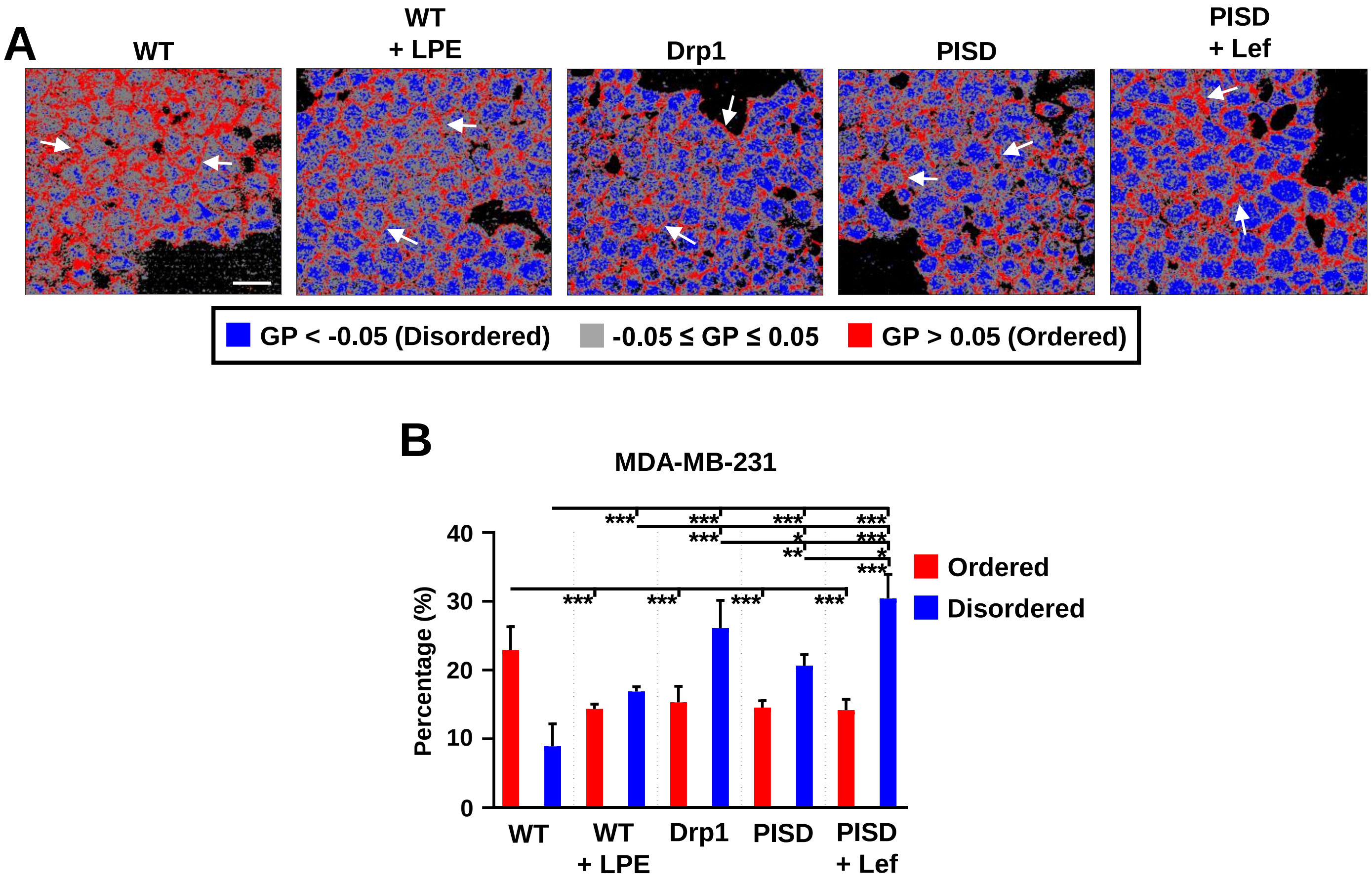

Supplemental Figure S3. Mitochondrial fission increases membrane disorder in MDA-MB-231 cells. A. Representative images of MDA-MB-231-WT, WT treated with α-lysophosphatidylethanolamine (LPE, 50 µM), PISD, PISD treated with leflunomide (Lef, 50 µM), or Drp1 cells stained with laurdan. Images show pseudocolored general polarization (GP) values. Images use the same red, blue, and grey color scheme described in **Figure 2E**. Arrows point to cell membranes. Scale bar = 50 µm. **B.** Graph shows mean + SD for the percentage of all ordered and disordered pixels for each image in (**A**) (n = 10 images per group). * = $p < 0.05$. ** = $p < 0.01$. *** = $p < 0.0001$. Data demonstrate that enforcing mitochondrial fission shifts to lower GP values, indicating reduced order of membranes.

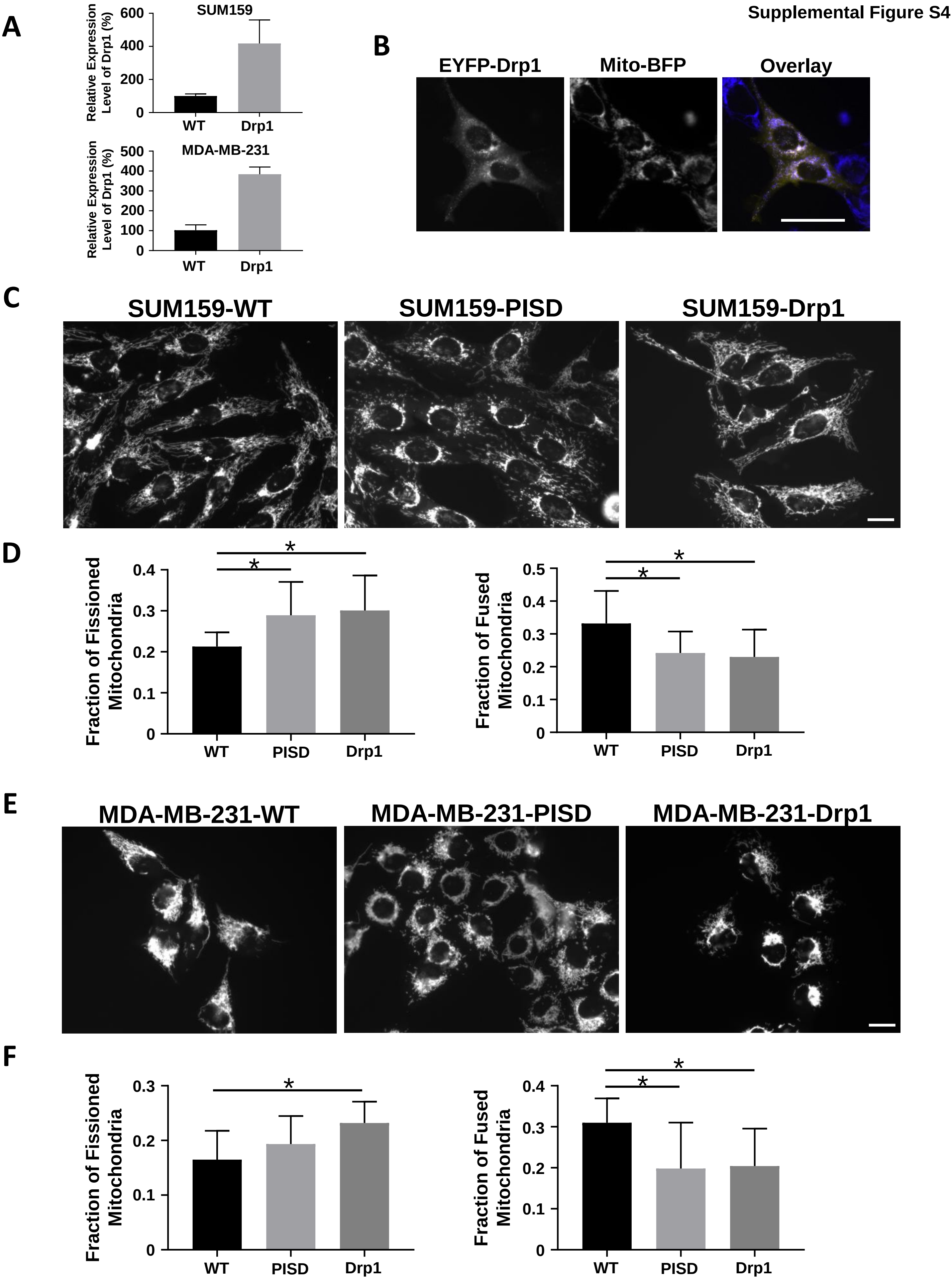
A
SUM159
Relative Expression Level of Drp1 (%)
600
400
200
0
WT
Drp1
MDA-MB-231
Relative Expression Level of Drp1 (%)
500
400
300
200
100
0
WT
Drp1
B
EYFP-Drp1
Mito-BFP
Overlay
C
SUM159-WT
SUM159-PISD
SUM159-Drp1
D
Fraction of Fissioned Mitochondria
0.4
0.3
0.2
0.1
0
WT
PISD
Drp1
*
*
Fraction of Fused Mitochondria
0.5
0.4
0.3
0.2
0.1
0
WT
PISD
Drp1
*
*
E
MDA-MB-231-WT
MDA-MB-231-PISD
MDA-MB-231-Drp1
F
Fraction of Fissioned Mitochondria
0.3
0.2
0.1
0
WT
PISD
Drp1
*
Fraction of Fused Mitochondria
0.4
0.3
0.2
0.1
0
WT
PISD
Drp1
*
*

Supplemental Figure S4. Drp1 drives mitochondrial fission. A. qRT-PCR results demonstrate > 3-fold overexpression of Drp1 in both SUM159 and MDA-MB-231 triple negative breast cancer cells. Graph shows mean values + SD expression relative to WT cells (n = 3). **B.** Representative images of EYFP-Drp1 transiently transfected into SUM159 cells stably expressing Mito-BFP. Scale bar is 20 µm. Images show localization of Drp1 (yellow) with mitochondria (blue). **C-F.** Representative fluorescence images of wild-type (WT), PISD, and Drp1 cells stably expressing mitochondrially-targeted BFP (Mito-BFP) demonstrating that PISD and Drp1 drive mitochondrial fission in SUM159 (**C**) and MDA-MB-231 (**E**) cells. Scale bar is 20 µm. Graphs show the mean fraction + SD of fissioned or fused mitochondria in SUM159 (**D**) and MDA-MB-231 (**F**) cells. (n ≥ 10 images for each group). * = $p < 0.05$.

Supplemental Figure S5

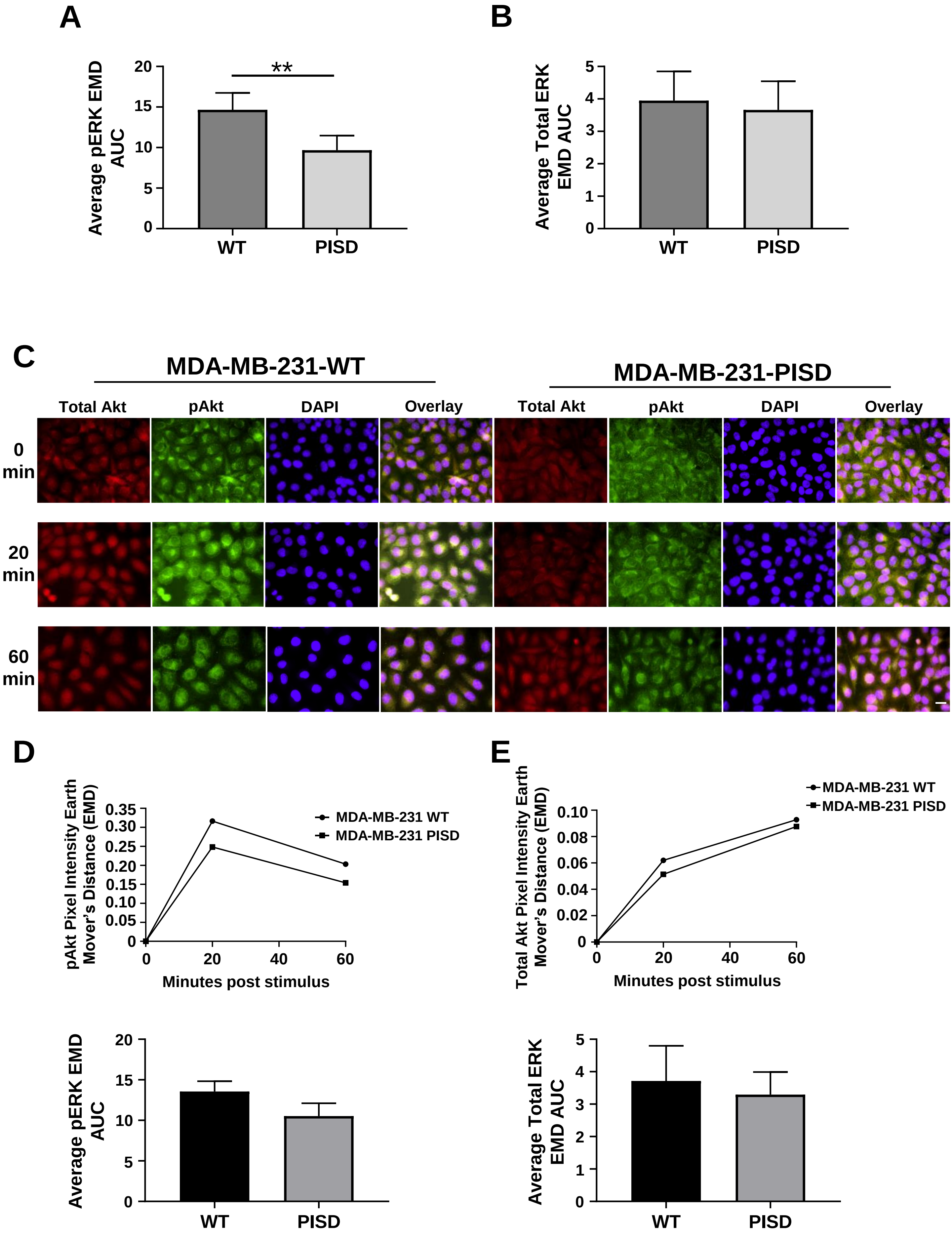

Supplemental Figure S5. PISD reduces ERK and Akt phosphorylation. A, B. Graphs show area-under-the-curve (AUC) for pERK (**A**) and total ERK (**B**) in SUM159-WT and PISD cells treated with serum (10%) in **Figure 3A** (n = 3). ** = $p < 0.05$. **C.** Representative overlaid images of immunofluorescence staining of total (red) Akt, phosphorylated (green) Akt, and DAPI (nuclei, blue) in MDA-MB-231-WT and PISD cells at the initial time point (0 min) and 20 and 60 minutes after addition of serum (10%). DAPI marks nuclei (blue). PISD cells exhibit reduced amplitude and duration of phosphorylated Akt (increased red in the overlaid images at 20 and 60 min time points). Scale bar is 20 µm. **D, E.** Graphs show earth mover's distance (EMD) and AUC of the green intensity (pAkt, **D**) or red intensity (total Akt, **E**) between each time point after addition of stimulus (10% serum) relative to the initial time point for Akt.

Supplemental Figure S6

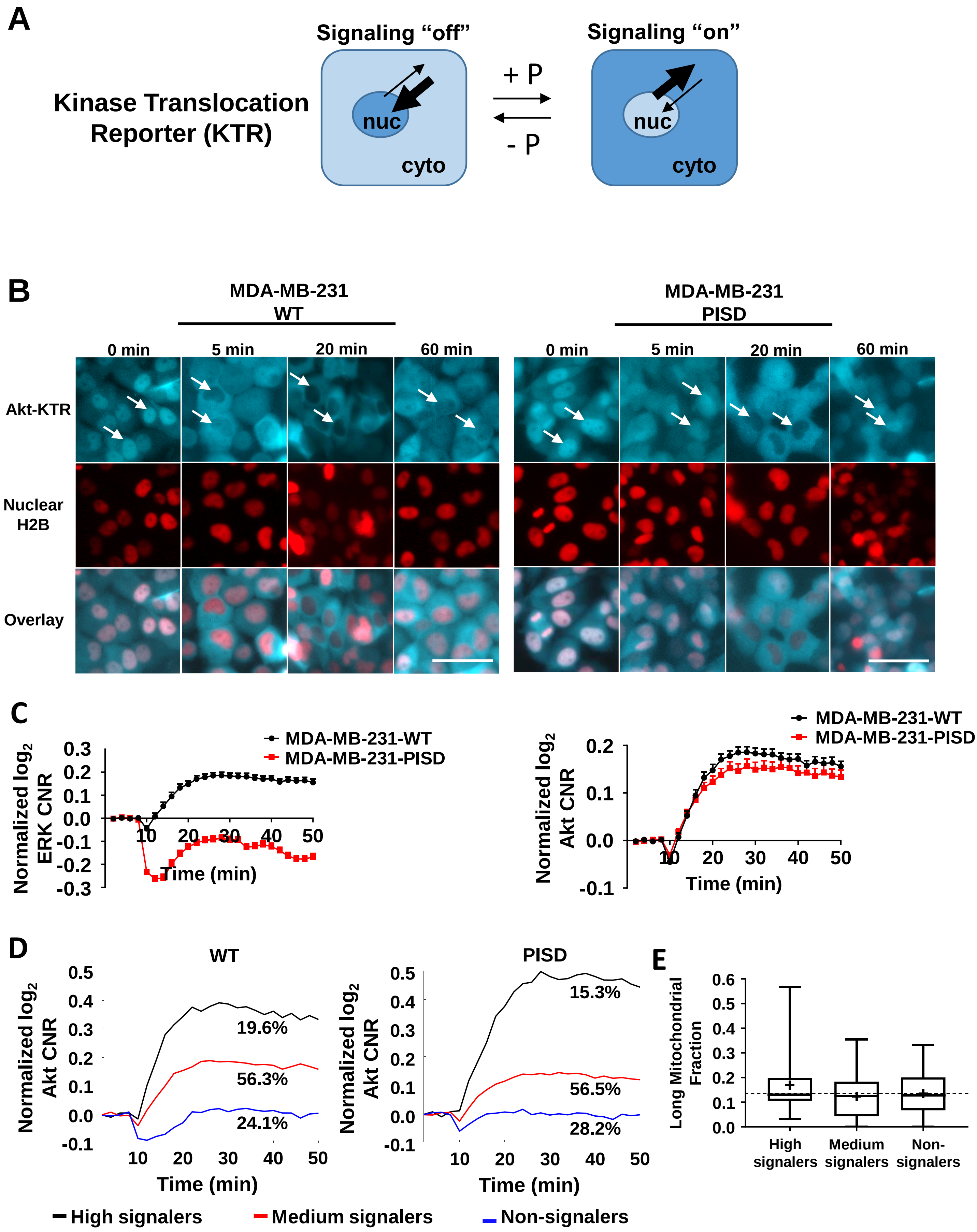

Supplemental Figure S6. Fissioned mitochondria reduce signaling output to Akt in single cells. A. Schematic representation of a kinase translocation reporter (KTR). Phosphorylation and dephosphorylation of the kinase substrate drives the reporter to the cytoplasm (signaling pathway “on”) or into the nucleus (signaling pathway “off”), respectively. **B.** Representative fluorescence images of Akt-KTR (cyan) and nuclear H2B-mCherry (red) in MDA-MB-231-WT and PISD cells at the initial time point (0 min) and 5, 20, and 60 minutes after addition of 10% serum show translocation of cyan fluorescence out of the nucleus (arrows, darker nucleus, Akt activation). The Akt-KTR shows enhanced Akt activation in WT compared with PISD cells, respectively. Scale bar is 50 µm. **C.** Graph shows normalized Akt and ERK activation in response to serum in an average MDA-MB-231-WT and PISD stably expressing cell. We normalized each point to the average CNR value of time points before serum addition. Graph shows mean + SEM. **D.** Graph shows mean normalized Akt activation in response to serum in high, medium, or non-signaler groups for MDA-MB-231-WT and PISD stably expressing cells. Percentages denote the percentage of cells that fall into the high, medium or non-signaling groups for each cell type. High, medium, and low signaling groups were determined by Akt AUC values that roughly equally split the high and low signaling groups in the WT cells. The same cutoff numbers were used for both WT and PISD cells. Each point is normalized to the average CNR value of time points before serum addition. **E.** Box plot and whiskers show the fraction of long (fused) mitochondria in high, medium, or non-signaling MDA-MB-231-WT cells (n = 122).

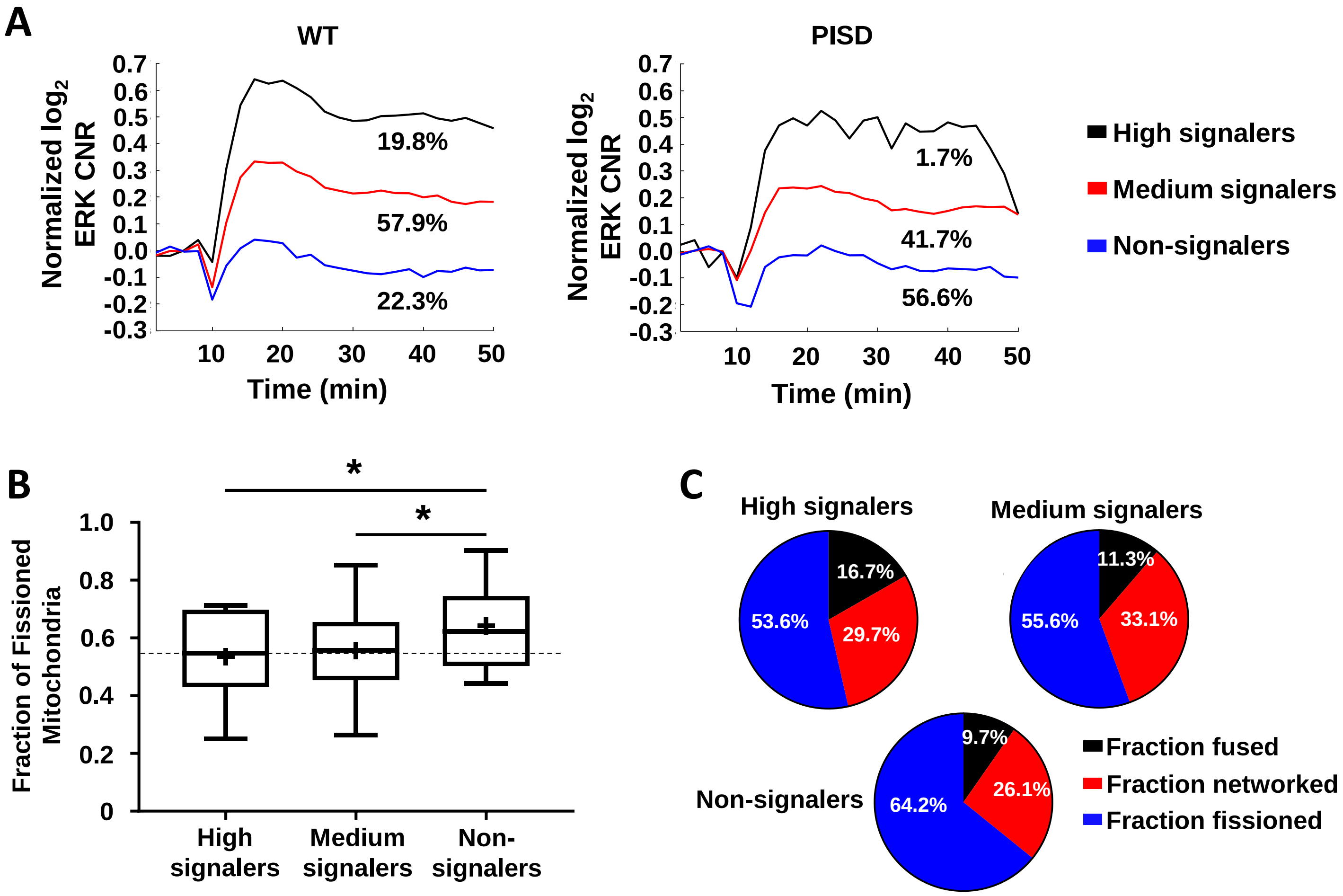

Supplemental Figure S7. PISD reduces the number of high signalers in SUM159 cells. A. Graphs showing average normalized ERK activation in response to serum in high, medium, or non-signaling groups for SUM159-WT and PISD stably expressing cells from **Figure 3B**. Percentages denote cells that fall into high, medium or non-signaling groups defined by ERK AUC values that split approximately equally the high and low signaling groups in the WT cells. We applied the same cutoff numbers for both WT and PISD cells. Each point is normalized to the average CNR value of time points before serum addition. **B.** Box plots and whiskers display the fraction of fissioned mitochondria for SUM159-WT cells with high, medium, and non-signaling responses of ERK following treatment with 10% serum. Line within the box denotes the median and the "+" symbol denotes the mean. Dashed line represents the median of the high signalers. High signalers: n = 18 cells, medium signalers: n = 57 cells, non-signalers: n = 21 cells. * = $p < 0.05$. **C.** Graphs show percentage of fused, networked, and fragmented mitochondria in SUM159-WT cells in high, medium, and non-signaling groups based upon ERK AUC in **Figure 3B**. Networked mitochondria are defined as having a major length axis less than 2 times the minor length axis. High signalers contain the most fused mitochondria and least fragmented mitochondria compared to medium and non-signalers, respectively.

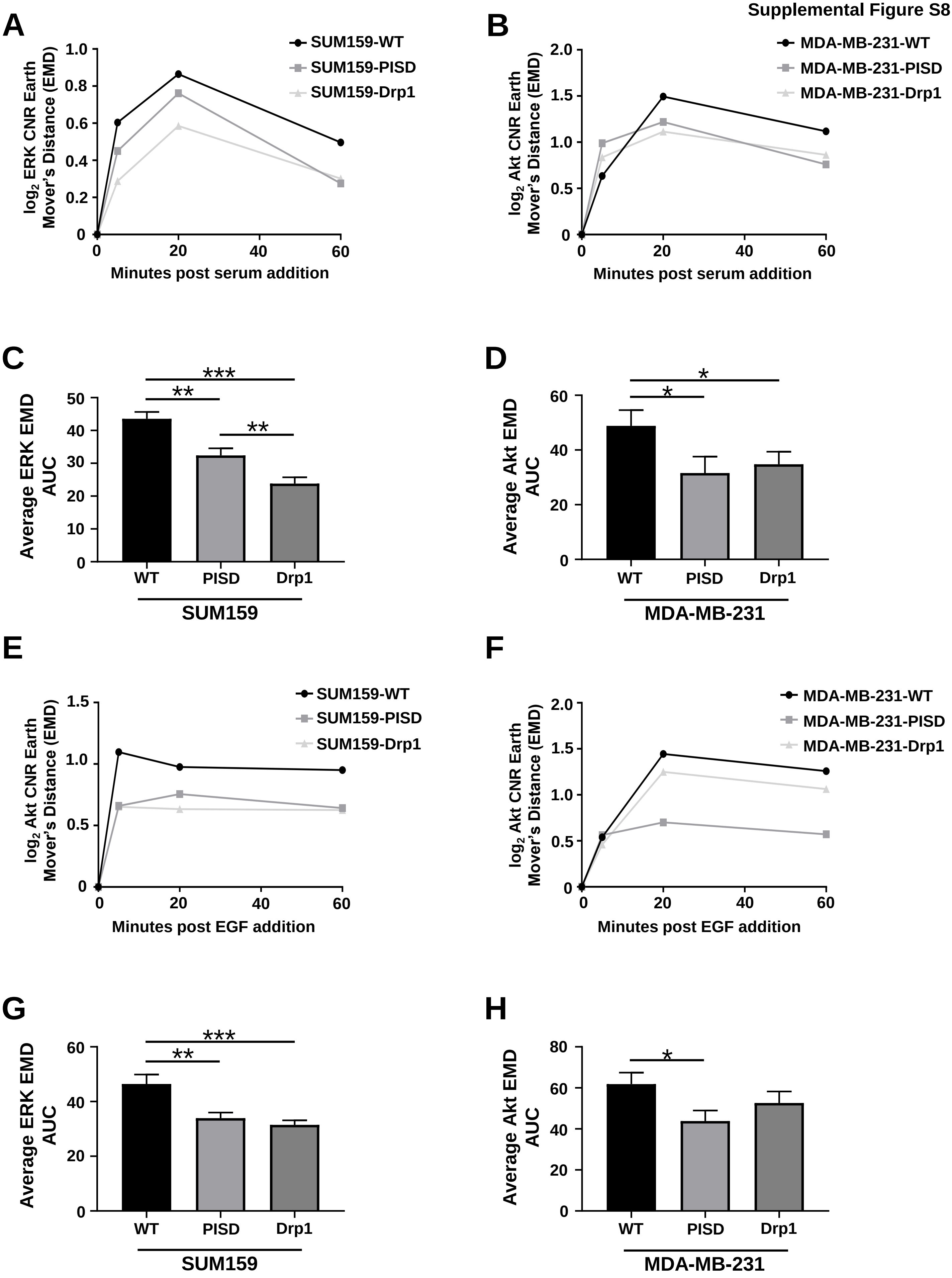
A
log2 ERK CNR Earth Mover's Distance (EMD)
Minutes post serum addition
SUM159-WT
SUM159-PISD
SUM159-Drp1
B
log2 Akt CNR Earth Mover's Distance (EMD)
Minutes post serum addition
MDA-MB-231-WT
MDA-MB-231-PISD
MDA-MB-231-Drp1
C
Average ERK EMD AUC
WT
PISD
Drp1
SUM159
D
Average Akt EMD AUC
WT
PISD
Drp1
MDA-MB-231
E
log2 Akt CNR Earth Mover's Distance (EMD)
Minutes post EGF addition
SUM159-WT
SUM159-PISD
SUM159-Drp1
F
log2 Akt CNR Earth Mover's Distance (EMD)
Minutes post EGF addition
MDA-MB-231-WT
MDA-MB-231-PISD
MDA-MB-231-Drp1
G
Average ERK EMD AUC
WT
PISD
Drp1
SUM159
H
Average Akt EMD AUC
WT
PISD
Drp1
MDA-MB-231

Supplemental Figure S8. Drp1 reduces ERK and Akt signaling. A-D. Earth mover's distance (EMD) and area-under-the-curve (AUC) between each time point after addition of stimulus (serum 10% (**A and B**) or EGF (50 ng/mL, **C and D**) relative to the initial time point for the Akt (right, SUM159 ≥ 455 cells) and ERK (left, MDA-MB-231 ≥ 123 cells) KTR. * = $p < 0.05$. ** = $p < 0.01$. *** = $p < 0.0001$. These data show that Drp1 inhibits ERK and Akt signaling.

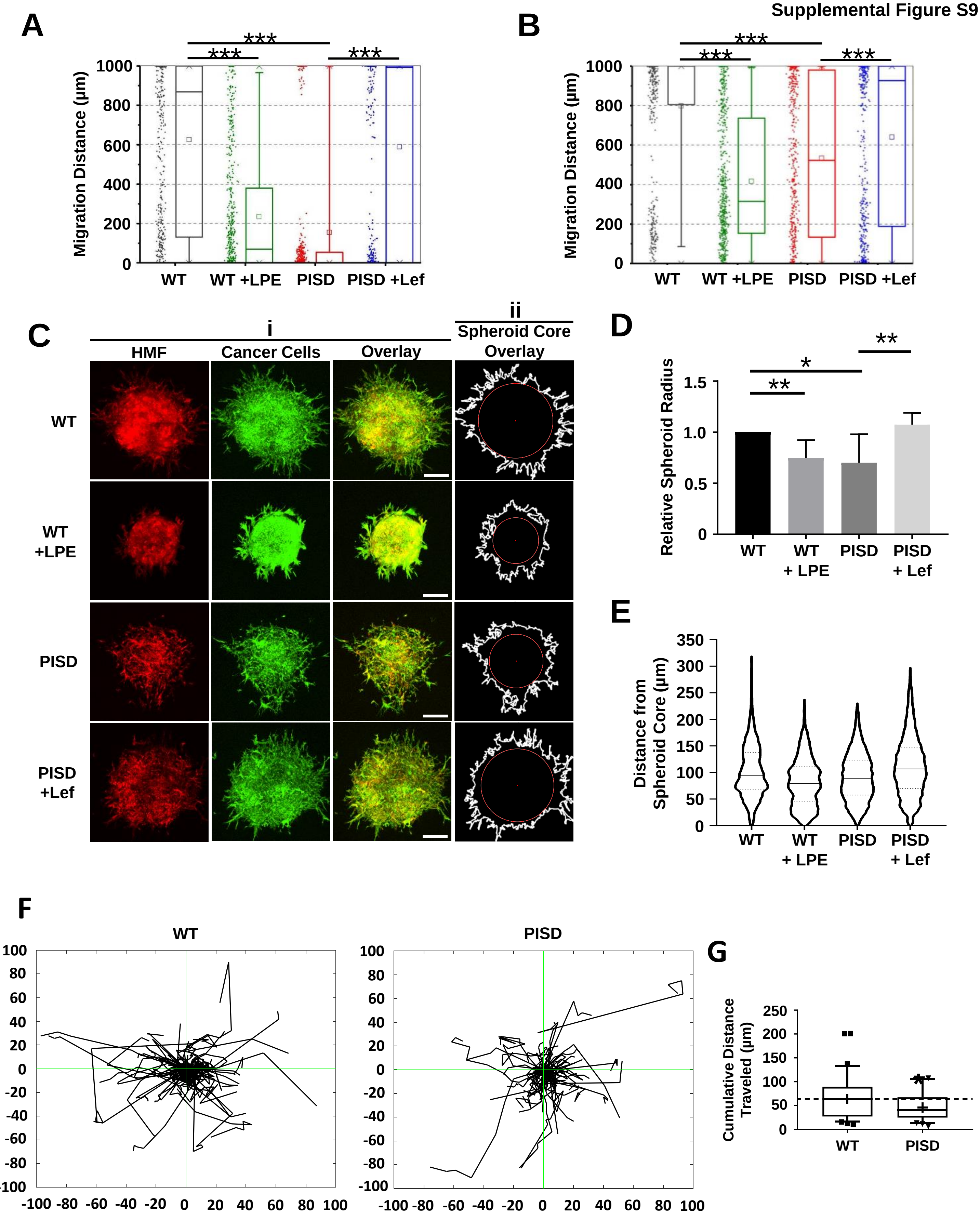
A
B
***
Migration Distance (µm)
1000
800
600
400
200
0
WT
WT +LPE
PISD
PISD +Lef
C
i
ii
HMF
Cancer Cells
Overlay
Spheroid Core Overlay
WT
WT +LPE
PISD
PISD +Lef
D
Relative Spheroid Radius
1.5
1.0
0.5
0
WT
WT + LPE
PISD
PISD + Lef
**
*
E
Distance from Spheroid Core (µm)
350
300
250
200
150
100
50
0
WT
WT + LPE
PISD
PISD + Lef
F
WT
PISD
100
80
60
40
20
0
-20
-40
-60
-80
-100
G
Cumulative Distance Traveled (µm)
250
200
150
100
50
0
WT
PISD

Supplemental Figure S9. Mitochondrial fission reduces cell migration. A, B. Graphs show box and whisker plot summaries and cell frequency distributions for migration of SUM159- (**A**) and MDA-MB-231- (**B**) WT and PISD cells treated with either vehicle control, LPE, or Lef toward serum in a microfluidic migration device (n = 600 cells). *** = $p < 0.0001$. **C. i.** Images of spheroids composed of SUM159-WT or PISD cells that express Mito-GFP (green) and human mammary fibroblasts (HMFs, red) two days after embedding into fibrin gels. **ii.** Example images define the perimeter of cancer cells in a spheroid (white line), center of the spheroid (red dot), and spheroid core (red circle, circle from the center of the spheroid with a radius of the distance from the center to the closest point on the perimeter) for each of the groups. Scale bar = 200 µm. **D.** Violin plot demonstrates the distribution of the distance each point on the perimeter of the spheroid from the spheroid core (n ≥ 4 spheroids per group, n ≥ 32,285 perimeter points per group). Solid line in the middle of the violin plot denotes the median. Dashed lines represent the upper and lower quartiles. These data show reduced migration in cells with greater mitochondrial fission (WT + LPE, PISD). **E.** Graph shows the mean radius of experimental spheroids relative to WT spheroids (n ≥ 4 spheroids per group). * = $p < 0.05$, ** = $p < 0.01$. **F.** Rose plots depict migratory distance traveled by single SUM159-WT (n = 83) or PISD (n = 105) cells on a tissue engineered construct with aligned engineered fibrillary fibronectin. Cells were imaged every 20 minutes for 3 hours. x- and y-axis display arbitrary units. **G.** Box and whisker plots for cumulative movement distance for SUM159-WT and PISD cells on scaffolds with aligned fibronectin fibers. Line within the box denotes the median and the “+” symbol is the mean SUM159-WT (n = 83) and PISD (n = 105) cells. Dashed line represents the median of the WT cells. * = $p < 0.05$.

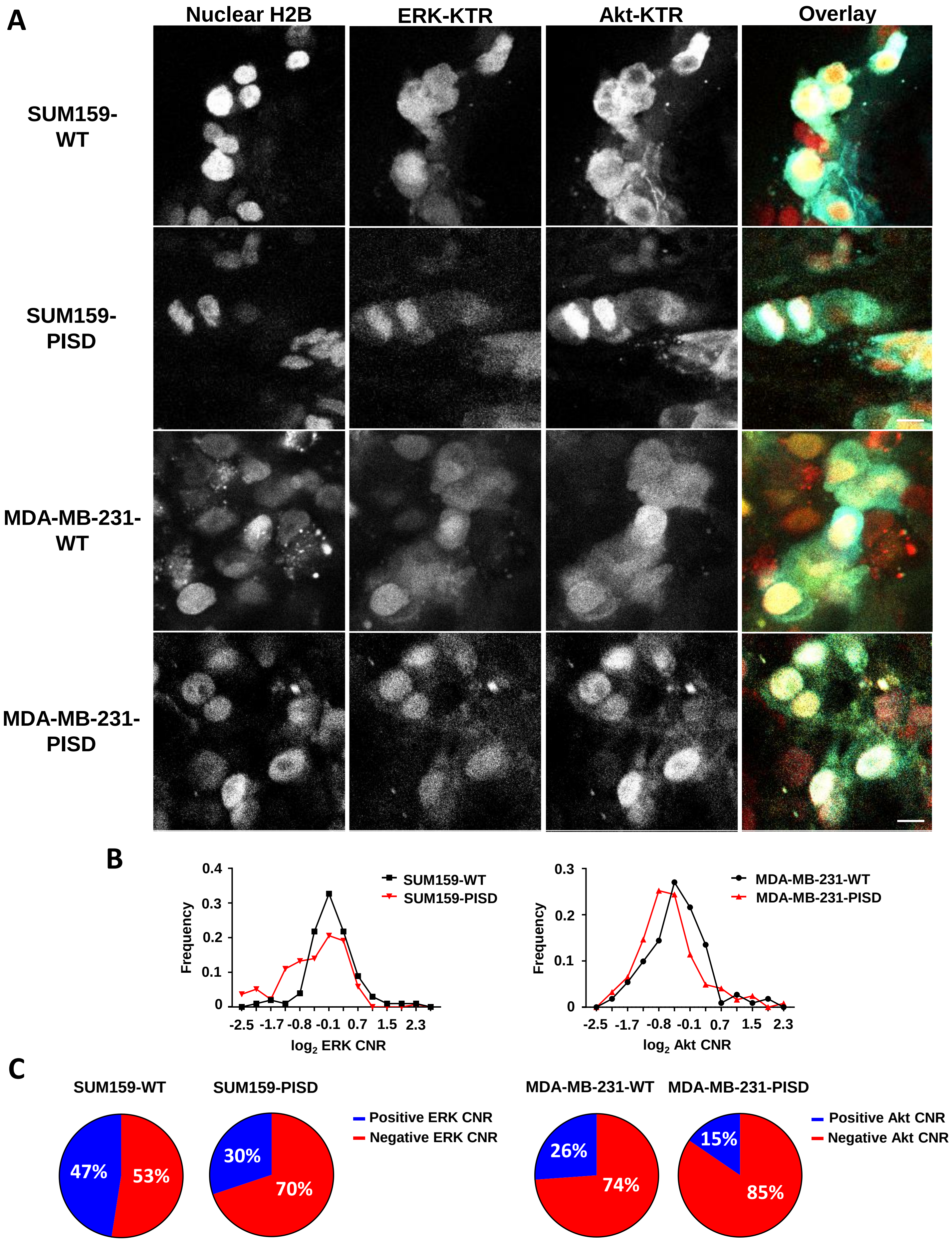
A
Nuclear H2B
ERK-KTR
Akt-KTR
Overlay
SUM159-WT
SUM159-PISD
MDA-MB-231-WT
MDA-MB-231-PISD
B
Frequency
0.4
0.3
0.2
0.1
0
-2.5 -1.7 -0.8 -0.1 0.7 1.5 2.3
log2 ERK CNR
SUM159-WT
SUM159-PISD
Frequency
0.3
0.2
0.1
0
-2.5 -1.7 -0.8 -0.1 0.7 1.5 2.3
log2 Akt CNR
MDA-MB-231-WT
MDA-MB-231-PISD
C
SUM159-WT
SUM159-PISD
47%
53%
30%
70%
Positive ERK CNR
Negative ERK CNR
MDA-MB-231-WT
MDA-MB-231-PISD
26%
74%
15%
85%
Positive Akt CNR
Negative Akt CNR

Supplemental Figure S10. PISD reduces oncogenic signaling in the bone marrow niche. A. Representative fluorescence images show ERK-KTR (yellow), Akt-KTR, (cyan), and nuclear H2B-mCherry (red) in bone marrow following femoral artery injection of SUM159- or MDA-MB-231-WT or PISD stably expressing cells. Scale bar is 10 µm. **B.** Histograms display frequency distributions for cytoplasmic-to-nuclear ratio (CNR) of fluorescence for MDA-MB-231- (left, Akt-KTR) and SUM159- (right, ERK-KTR) WT and PISD cells. A shift to the left indicates a reduction in kinase signaling. **C.** Graphs show percentages of positive (> 0) and negative (< 0) Akt-KTR (MDA-MB-231, left) and ERK-KTR (SUM159, right) CNR values for WT and PISD cells in bone marrow. Data show that in addition to reduced bone destruction shown in **Figure 1B**, mitochondrial fission blunts ERK and Akt signaling in the bone marrow niche.
